# Arginase-induced cell death pathways and metabolic changes in cancer cells are not altered by insulin

**DOI:** 10.1038/s41598-024-54520-z

**Published:** 2024-02-19

**Authors:** Hui Yi Chew, Goran Cvetkovic, Slobodan Tepic, James W. Wells

**Affiliations:** 1 Faculty of Medicine, Frazer Institute, The University of Queensland, 37 Kent Street, Brisbane, QLD 4102 Australia; 2 Hepius Biotech AG, Zurich, Switzerland

**Keywords:** Breast cancer, Cancer metabolism, Lung cancer

## Abstract

Arginine, a semi-essential amino acid, is critical for cell growth. Typically, de novo synthesis of arginine is sufficient to support cellular processes, however, it becomes vital for cancer cells that are unable to synthesise arginine due to enzyme deficiencies. Targeting this need, arginine depletion with enzymes such as arginase (ARG) has emerged as a potential cancer therapeutic strategy. Studies have proposed using high dose insulin to induce a state of hypoaminoacidaemia in the body, thereby further reducing circulating arginine levels. However, the mitogenic and metabolic properties of insulin could potentially counteract the therapeutic effects of ARG. Our study examined the combined impact of insulin and ARG on breast, lung, and ovarian cell lines, focusing on cell proliferation, metabolism, apoptosis, and autophagy. Our results showed that the influence of insulin on ARG uptake varied between cell lines but failed to promote the proliferation of ARG-treated cells or aid recovery post-ARG treatment. Moreover, insulin was largely ineffective in altering ARG-induced metabolic changes and did not prevent apoptosis. In vitro, at least, these findings imply that insulin does not offer a growth or survival benefit to cancer cells being treated with ARG.

## Introduction

Arginine is an intermediate metabolite in the urea cycle and key for the production of nitric oxide and polyamines to support cell growth^1,2^. Arginine is considered to be a semi-essential amino acid as the systemic requirements can be supplemented by the intestinal-renal axis^3^. However, some cancer cells are dependent on extracellular sources of arginine due to rapid growth requirements or an inability to synthesise arginine due to a lack of argininosuccinate synthetase (ASS1) expression^2,4^. These cells are auxotrophic for arginine, and depletion of arginine using arginine-depleting enzymes such as arginase (ARG) and arginine deaminase (ADI) has been proposed as a form of cancer therapy^5–8^.

In the body, arginine can be replenished from several sources including the diet, de novo synthesis of arginine from citrulline mediated by ASS1 and argininosuccinate lyase and release from cellular structures through processes of protein turnover such as cellular autophagy and muscle catabolism^2,9–11^. To prevent muscle catabolism that could increase circulating arginine, several groups have proposed using high dose insulin to induce hypoaminoacidaemia to decrease the baselines of amino acid by enhancing the uptake of branched-chain amino acids (valine, leucine and isoleucine) into cells^10,11^. This could also allow arginase to work to its full capability of depleting arginine as the arginase activity can be impacted through the actions of these branched-chain amino acids, which compete with arginine for the catalytic site of arginase^12^.

On the other hand, insulin is a growth factor that is able to activate the insulin receptor, leading to mitogenic effects on cells^13^. Cancer cells often overexpress the insulin receptor and insulin has been shown to directly stimulate cancer cell growth in vitro^13^. Thus, an excess of insulin could potentially be beneficial to cancer cell growth.

In this study, we sought to determine whether the addition of insulin would confer any potential proliferation and/or survival advantages to cancer cells being treated with ARG. We investigated and compared the effects of ARG and insulin treatment to ARG treatment alone in three different cancer types, namely breast, lung and ovarian cancer. First, we examined the cellular uptake of ARG and whether it was associated with cancer cell proliferation. Second, we looked at the metabolic effects induced by these treatments, and third, we investigated the types of cell death that are triggered by arginine depletion. We found that the addition of insulin to ARG treatment at both low and high doses did not prevent the effects that arginine depletion has on cancer cells in vitro.

## Results

### Effect of insulin on ARG uptake into cells

**Figure 1:**
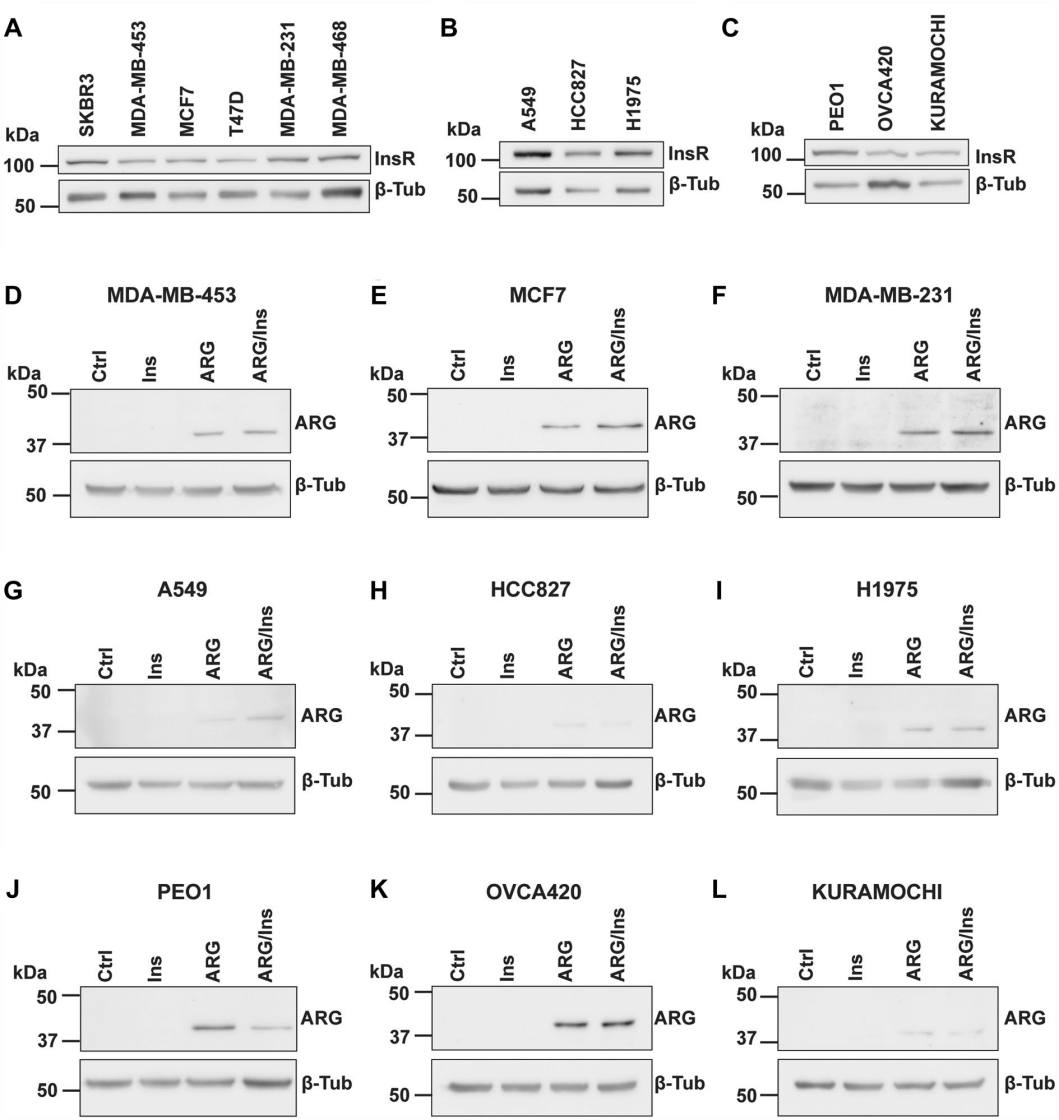
Western blot analysis of insulin receptor (**A**–**C**) and arginase (**D**–**L**) in the indicated cancer cell lines. β-Tubulin was used as the loading control. Representative cropped images of two independent experiments with similar results are shown. Un-processed blots are available in Supplementary Figures S2–S13.

To investigate the potential effects of insulin on ARG treatment, we first determined the expression of insulin receptor on six breast cancer cell lines (Fig. 1A), three lung cancer cell lines (Fig. 1B), and three ovarian cancer cell lines (Fig. 1C). Western blot analysis showed that all cancer cell lines used in this study expressed various levels of insulin receptor (see Supplementary Fig. S1A–C for quantitation, and Supplementary Fig. S2–S4 for unprocessed images). Out of the six breast cancer cell lines, three were chosen for further analysis (one from each breast cancer subtype, namely MDA-MB-453 (HER2^+^), MCF7 (ER+ /PR^+^), and MDA-MB-231 (TNBC)).

Next, we investigated whether insulin affects exogenous ARG uptake into cells. Western blot analysis showed that arginase was only detectable when exogeneous ARG was added to the treatment as no band was observed for both the control and insulin only treatment groups. Two breast cancer cell lines (MCF7 and MDA-MB-231) showed an increase in ARG level when treated with the combination of ARG and insulin, however the remaining seven cell lines showed either a marginal effect, or a decrease in ARG level when compared to ARG treatment alone (Fig. 1D–I; see Supplementary Fig. S1D–L for quantitation, and Supplementary Fig. S5–S13 for unprocessed images). The effects of insulin on exogenous ARG uptake did not appear to be associated with the level of insulin receptor expression, for example, MDA-MB-231 cells and PEO1 cells both have high levels of insulin receptor expression, but their ARG levels following co-incubation with insulin were diametrically opposed (Supplementary Fig. S1).

### Insulin does not confer any proliferative advantages to cancer cell lines treated with ARG

**Figure 2:**
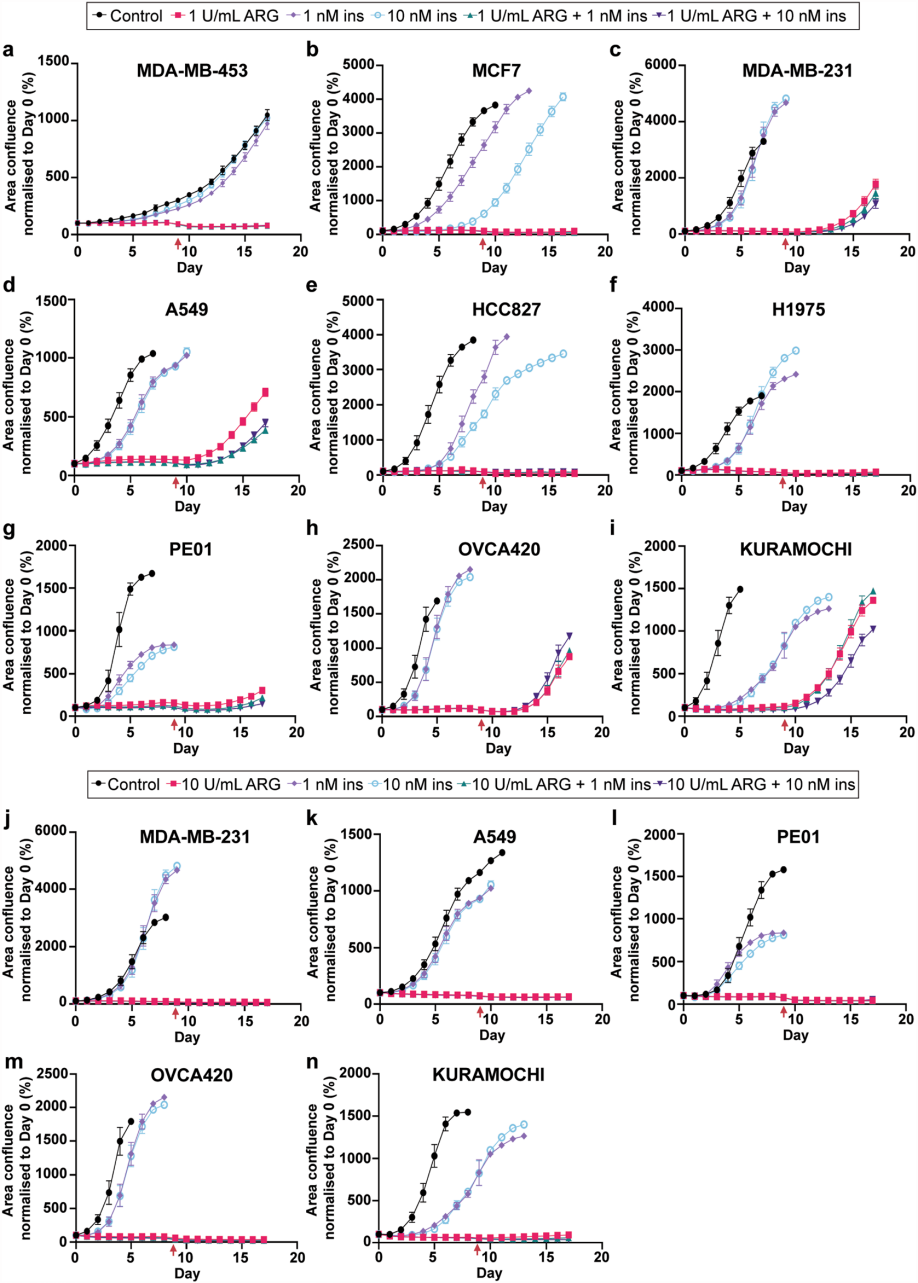
Assessment of arginase and insulin on the growth and recovery of breast, lung, and ovarian cancer cell lines. Cell lines were treated with 1U/ml ARG with/without 1nM and 10nM of insulin for 9 days, and changed to complete media (indicated by the red arrow) for the following 8 days (**a**–**i**). (**j**–**n**) Cell lines that were able to recover from 1U/ml ARG treatment were subjected to 10U/ml ARG with/without 1nM and 10nM of insulin. Representative data are shown from two independent experiments with similar results. Data are presented as mean ± S.D, and are normalised to Day 0.

Since the combination of ARG and insulin showed differential ARG uptake patterns between the cancer cell lines, we were interested to determine if that would have any impact on cell proliferation and most importantly, if cells were able to recover following ARG treatment. Cells were treated for nine days with ARG alone or ARG/ insulin and then treatment media was exchanged for complete media and recovery was monitored for a further eight days. Cells were also treated with low (1 nM) and high (10 nM) doses of insulin to define the relevance of insulin dose. In all cell lines tested, the addition of 1 nM or 10 nM insulin either did not affect cell growth, or delayed cell growth compared to the control group (MnCl_2_ alone; Fig. 2). Out of the three breast cancer cell lines, only MDA-MB-231 was able to recover following treatment with ARG, and recovery appeared unaffected by insulin (Fig. 2c). Similarly, A549 was the only lung cancer cell line that recovered following ARG/insulin treatment (Fig. 2d). However, all three ovarian cancer cell lines recovered following ARG/insulin treatment (Fig. 2g–i). The four cell lines that were susceptible to ARG alone remained susceptible when insulin was present, suggesting that the addition of insulin to ARG treatment did not confer any proliferation advantages to these cells.

We also subjected the five cell lines that were able to recover after treatment with low dose of ARG to high dose of ARG (10 U/mL). None of the cell lines were able to recover following treatment, and again insulin did not appear to affect sensitivity to ARG (Fig. 2j–n).

### ARG treatment induces global metabolic changes

To understand the effects that ARG and insulin have on the metabolomic landscape of the cancer cells, we picked four cell lines, MDA-MB-231, A549, H1975 and KURAMOCHI, and treated them with low and high doses of ARG with/without 1 nM insulin. Amino acid analysis was then performed on cell lysates. The level of arginine in ARG-containing groups was consistently below the level of arginine in the control group, confirming ARG activity, and the addition of insulin to ARG did not influence the level of arginine, ornithine, or citrulline (Supplementary Fig. S14).

**Figure 3:**
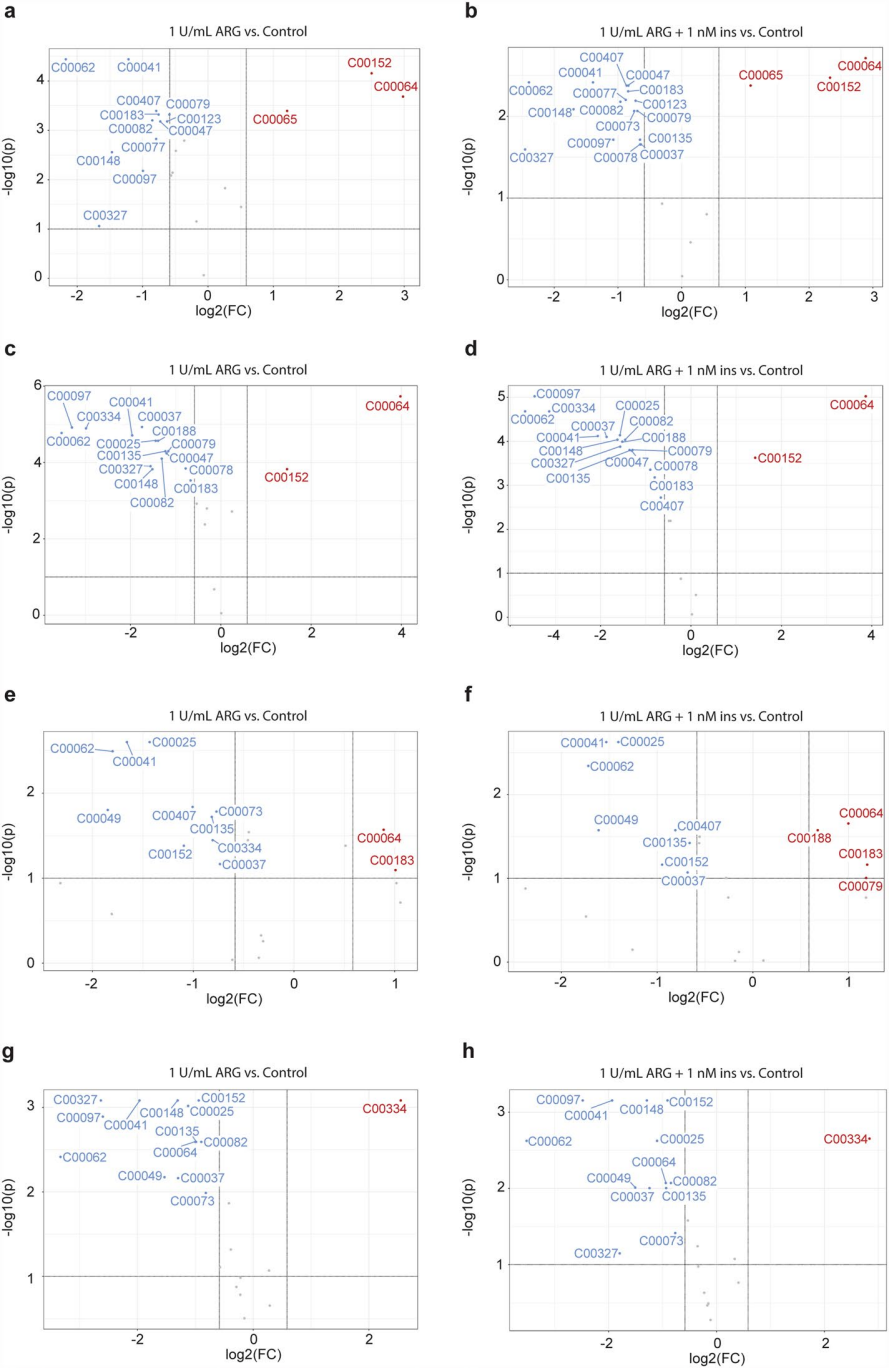
Amino acid analysis of cell lysates following treatment with ARG or ARG and insulin. Left: Cells treated with 1U/mL ARG; Right: Cells treated with 1U/mL ARG + 1nM insulin (ins). (**a**, **b**) MDA-MB-231 cell lysates, (**c**, **d**) A549 cell lysates, (**e**, **f**) H1975 cell lysates and (**g**, **h**) KURAMOCHI cell lysates. Volcano plots generated using MetaboAnalyst 5.0. Fold-change (FC) threshold was set as 1.5 and *p*-value (generated from the t-tests) threshold was set as 0.1 FDR. KEGG ID’s are shown (described fully in Supplementary Table 1). Red dots indicate significant increases, blue dots indicate significant decreases, and grey dot indicates not significant.

Univariate statistical tests were used to identify the amino acids that showed significant changes in treatment groups compared to control (Fig. 3, Supplementary Table S1–4). When compared to the control group, ARG treatment caused an increase in several metabolites. However, the addition of insulin did not alter the metabolite pattern induced by ARG for each cell line, with the exception of H1975 cells, where an increase in threonine and phenylalanine was also observed (Fig. 3e,f). Furthermore, the metabolites increased by ARG were relatively inconsistent between cell lines, with the exception of glutamine, which was increased in all cell lines except for KURAMOCHI cells, and asparagine, which was increased in both MDA-MB-231 and A549 (Fig. 3). The addition of insulin, however, did increase the number of metabolites that were decreased following ARG treatment in both MDA-MB-231 and A549 cell lines. We observed a decrease in histidine, glycine, methionine, and tryptophan in MDA-MB-231 cells (Fig. 3a–b), and a decrease in isoleucine in A549 cells (Fig. 3c,d).

### Changes in metabolites had a profound impact on metabolic pathways

**Figure 4:**
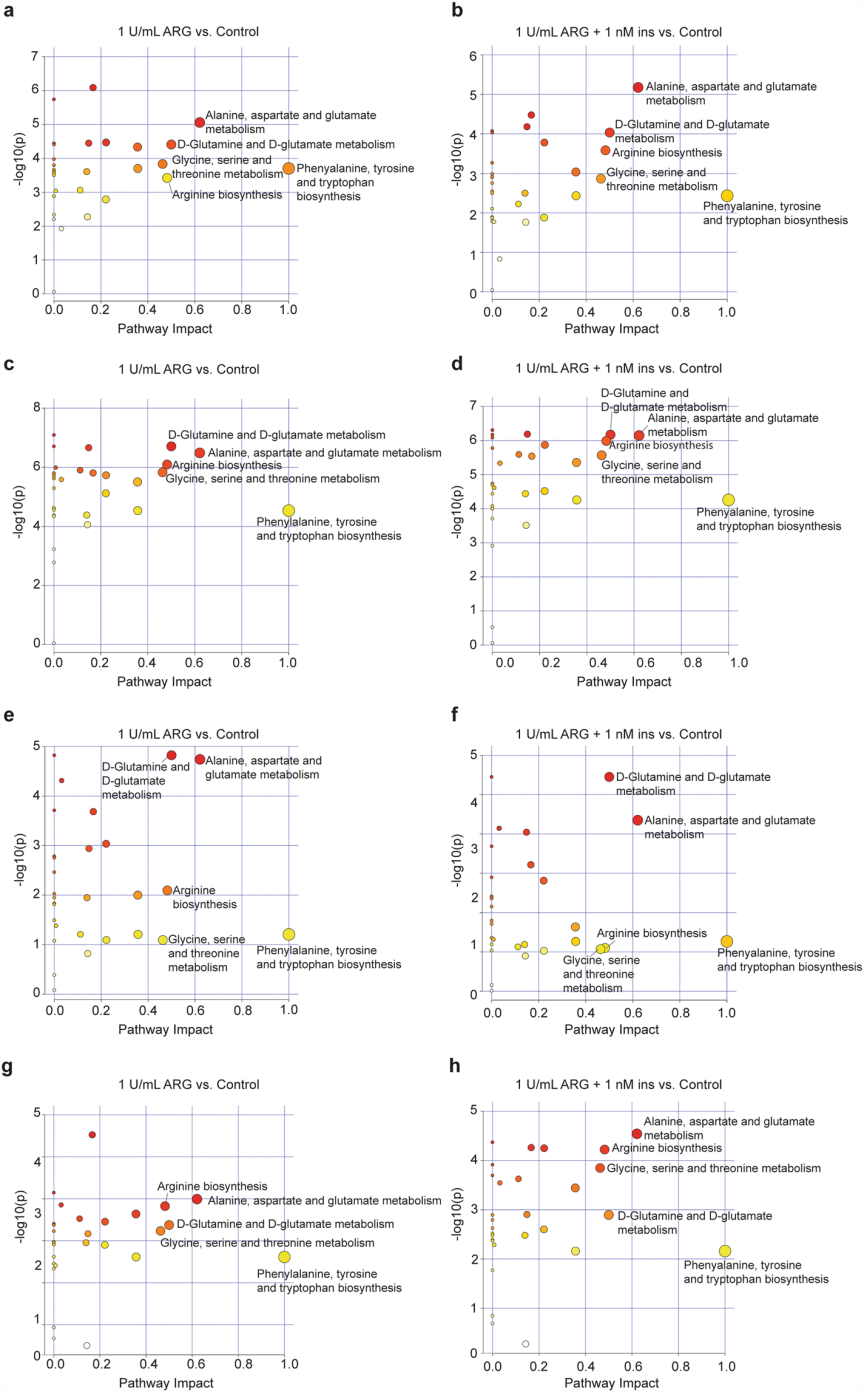
Pathway analysis of differentially expressed metabolites in cancer cell lysates following treatment with ARG or ARG and insulin. Amino acid levels were subjected to MetPA analysis and presented as pathway impact value vs. *p*-value. The size and colour of each dot correlates with its pathway impact and *p*-value (red > orange > yellow), respectively. Left: Cells treated with 1U/mL ARG; Right: Cells treated with 1U/mL ARG + 1nM ins. (**a**–**b**) MDA-MB-231 cell lysates, (**c**–**d**) A549 cell lysates, (**e**–**f**) H1975 cell lysates and (**g**–**h**) KURAMOCHI cell lysates.

MetPA analysis was performed to further understand how the changes in metabolites observed in each cell line effect biological pathways. This analysis allows us to identify the most relevant biological pathways that are enriched between the control and treatment groups, as indicated by the pathway impact (Fig. 4, Supplementary Table S5–S16). Pathway impact is a general score demonstrating how much a pathway is perturbed by the metabolites. The size of the dots corresponds to the pathway impact score (the larger the circle, the more perturbed the corresponding pathway is) and the colour of the dots indicates significance (red being the most significant, yellow being the least significant). Across all four cell lines (MDA-MB-231, A549, H1975 and KURAMOCHI), the five biological pathways observed in cells treated with ARG were the same as those observed in cells treated with the ARG and insulin combination (Fig. 4a–h; Pathway impact > 0.4).

Thus, the addition of insulin to ARG treatment did not appear to cause any drastic change in biological pathways compared to ARG treatment alone. Furthermore, these pathways were also unaffected by ARG dose (Supplementary Fig. S15), suggesting that the increased sensitivity of cells to high dose ARG is not associated with metabolic differences.

### ARG induces apoptosis but not autophagy in cancer cell lines

**Figure 5:**
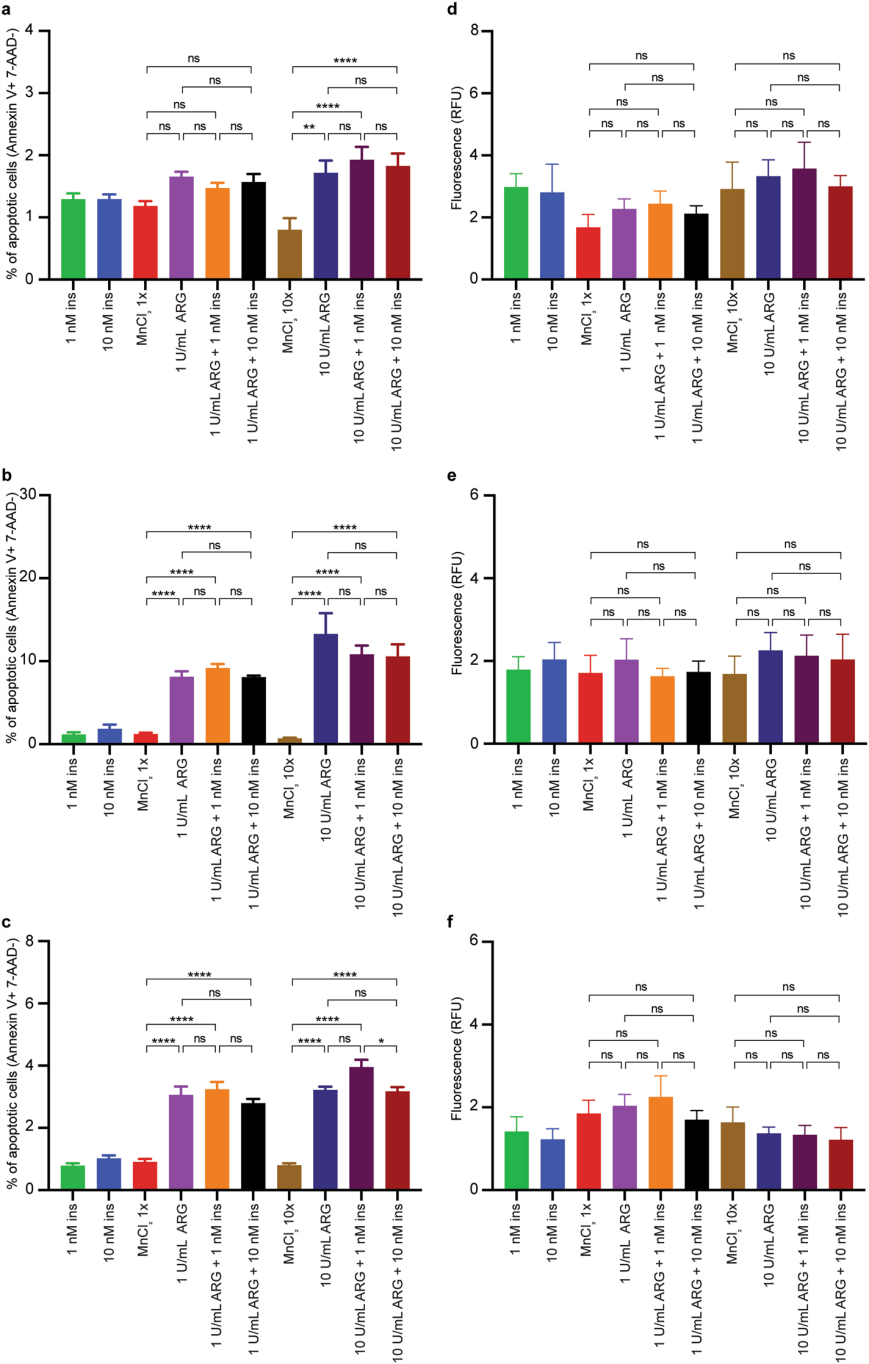
Apoptosis and autophagy analysis following treatment with ARG or ARG and insulin. (**a**–**c**) Apoptosis analysis of MDA-MB-231 (**a**), H1975 (**b**), and KURAMOCHI (**c**) cells by flow cytometry. Apoptotic cells were determined as being Annexin V^+^/7-AAD^−^. (**d**–**f**) Autophagy analysis of MDA-MB-231 (**d**), H1975 (**e**), and KURAMOCHI. Data are pooled from 3 independent experiments and are presented as mean ± S.E.M. One-way ANOVA followed by Tukey’s multiple comparisons test was used to determine *p*-value; ns p > 0.05, *p ≤ 0.05, **p ≤ 0.01, ****p ≤ 0.0001.

To elucidate the mechanism(s) through which ARG causes cell death, MDA-MB-231 and KURAMOCHI cells, which were resistant to 1 U/mL ARG, and H1975 cells, which were susceptible to 1 U/mL ARG were chosen for apoptosis and autophagy analyses (Fig. 5). Using flow cytometry, cells undergoing apoptosis were identified as Annexin V^+^/7-AAD^−^. After 72 h of culture, no significant differences were observed in MDA-MB-231 cells between MnCl_2_ control and 1 U/mL ARG treatment groups, however, significant differences were observed in H1975 and KURAMOCHI cells (Fig. 5a–c). No significant differences were observed in all cell lines when comparing between ARG treatment alone and ARG treatment with both doses of insulin. At 10 U/mL ARG, significant increases in apoptosis were observed compared to MnCl_2_ controls in all cell lines (Fig. 5a–c). Similar to low dose treatment groups, no significant differences were observed between ARG treatment alone and treatment groups of ARG and both doses of insulin in all cell lines (Fig. 5a–c, with the exception of 10 U/mL ARG+ 1 nM insulin vs. 10 U/mL ARG+ 10 nM insulin in KURAMOCHI cells; Fig. 5c). Overall, the data suggest that the addition of insulin to ARG treatment does not rescue cells from apoptotic cell death.

Autophagic vacuole staining did not reveal any differences between insulin only control, MnCl_2_ only control, or ARG treatment alone or combination treatments of ARG and insulin in any cell lines (Fig. 5d–f). Furthermore, a similar pattern was observed following treatment with both low and high doses of ARG, suggesting that ARG did not enhance autophagy.

## Discussion

Metabolic therapy with enzymes that deplete the amino acid arginine, are in early phase I and II clinical trials for the treatment of malignant melanoma, metastatic uveal melanoma, and hepatocellular carcinoma^5,7,8^. These tumour types are frequently reported to lack ASS1 expression, the rate-limiting enzyme for the de novo biosynthesis of arginine^14^. When exogenous arginine is depleted, for example by recombinant human ARG, the lack of arginine to support cellular metabolism and growth causes tumour cells to undergo dual-phase cell cycle arrest and concurrent apoptosis^15^. Healthy cells, in contrast, arrest their cell cycle in G_1_ and enter into a quiescent state from which they can fully recover following the reintroduction of arginine^16^. Early indications are that ARG injection has the potential to induce complete remission in melanoma patients whose cancers have progressed on current standard of care, including immunotherapy^6,7^.

Albeit at an early stage of clinical development, it is becoming apparent that the use of arginase injection alone may only induce complete remission in a small proportion of patients. Unlike in vitro studies in the laboratory, in which conditions leading to complete arginine depletion can be well-controlled, achieving complete and sustained arginine depletion in vivo is more challenging as it can be replenished from several sources such as through diet, de novo synthesis of arginine from citrulline, and release from cellular structures through processes of protein turnover, including systemic autophagy and muscle catabolism^9^. Perhaps unsurprisingly, the duration of arginine depletion was found to be crucial for clinical benefit in a phase II clinical trial of recombinant human ARG for the treatment of hepatocellular carcinoma^5^.

Insulin is an anabolic hormone used clinically to reduce blood glucose levels in patients with diabetes by enhancing glucose uptake and metabolism in cells. However, insulin has long been known to reduce circulating levels of amino acids, including arginine and branched-chain amino acids, in the blood^10,17^. The hypoaminoacidaemic effect of insulin is thought to be due to the promotion of amino acid uptake into cells and inhibition of muscle breakdown^10^. More recently, insulin has also been shown to reduce branched-chain amino acids in the blood through the induction of branched-chain a-ketoacid dehydrogenase in the liver^18^, and to inhibit autophagy through the inactivation of the Forkhead box O family of transcription factors and the autophagy-related protein complex, unc-51 like kinase 1 (ULK1)^19^. Therefore, through separate and complementary mechanisms, the inclusion of insulin into ARG treatment regimens has genuine potential to increase the efficacy of arginine depletion in vivo and warrants further investigation to determine whether combination with ARG affects the ability of ARG to induce cancer cell death.

Very little is known about the effect of insulin on the uptake of metabolic enzymes into cells. Our data suggest that different cancer cell lines exhibit differences in cellular uptake of exogenous ARG when cells were treated with both ARG and insulin simultaneously, which did not appear to be associated with the level of insulin receptor expression. This data suggests that insulin is unlikely to play a direct role in regulating the uptake of ARG into cancer cells.

Previous studies have shown that insulin binds to the insulin receptor, which activates signalling pathways such as the phosphatidylinositol 3-kinase (PI3K)/Akt pathway, and the Ras-mitogen-activated protein kinase (MAPK) pathway^20^. Both PI3K/Akt and MAPK pathways promote cell proliferation and survival by inhibiting pro-apoptotic proteins and stimulating other proteins and transcription factors, such as mammalian target of rapamycin complex 1 (mTORC1) and Elk1, that promote protein synthesis and transcription of survival genes, respectively ^20–22^.

In our studies however, four out of nine cell lines were unable to recover following treatment with low dose ARG (1 U/mL), and the remaining five cell lines were resistant to low dose ARG treatment, yet the addition of insulin during treatment did not impact upon cellular resistance nor recovery. Similarly, when the five cell lines resistant to low dose ARG were treated with high dose ARG (10 U/mL), the presence of insulin in the culture did not facilitate their recovery. Thus, the data suggest that insulin does not positively or negatively affect the impact of ARG on cancer cell growth.

Arginine is a versatile amino acid that is involved in numerous pathways, including protein synthesis and immune system regulation^23^. It is also one of the three amino acids that is able to directly activate mammalian target of rapamycin complex 1 (mTORC1) by binding to cellular arginine sensor for mTORC1 (CASTOR1)^24–26^. mTORC1 plays a central role in regulating cell growth and metabolism by sensing nutrient availability and cellular stress in order to maintain cellular homeostasis^27^. Under growth-optimal conditions, mTORC1 activates protein and nucleotide biosynthesis pathways, and inhibits autophagic pathways thereby preventing cell catabolism^27^. Similarly, under starvation conditions, mTORC1 regulates survival pathways, such as by inducing autophagy to compensate for nutrient deprivation.

Arginine deprivation has been shown by others to induce a metabolic shift in MDA-MB-231 cells^28^. When these cells were cultured in arginine-free media they were found to induce asparagine synthetase (ASNS), which resulted in increased aspartate consumption as aspartate was converted into asparagine, and an increase in glutamine due to a dysfunctional tricarboxylic acid (TCA) cycle^28^. Rather than using arginine-free media, we depleted arginine using ARG, which would have increased levels of ornithine and urea and could have triggered a different series of metabolic events. Furthermore, the presence of insulin could have contributed to PI3K/Akt/mTORC1 pathway activation^20^. We performed amino acid analysis on cancer cell lysates and included three resistant cell lines, MDA-MB-231, A549, and KURAMOCHI, and one sensitive cell line, H1975. Our data confirmed that arginine was significantly decreased when cells were treated with ARG and found that there were no significant differences in arginine levels when ARG treatment alone was compared to combined ARG and insulin treatment. Furthermore, levels of ornithine (which can be converted to citrulline through the urea cycle) and citrulline were not increased when comparing the cell lysates following three days of ARG or ARG/insulin treatment. In fact, a significant decrease in ornithine in MDA-MB-231 cells and a significant decrease in citrulline was observed in all three resistant cell lines. This may suggest that metabolic pathways had been altered, and indeed, it has been shown previously that in addition to glycolysis, cancer cells can also reactivate metabolic pathways including nucleotide and protein synthesis^29^, and inhibit mitochondrial metabolism^30^. Cancer cells starved of arginine are known to display a perturbed mitochondrial and TCA cycle ^28^. Dysfunctional mitochondria could potentially affect the conversion of ornithine to citrulline since that process happens in the mitochondria^31^. Furthermore, the urea cycle is also closely linked to the TCA cycle as the TCA cycle produces oxaloacetate, which gets converted to aspartate that gets utilised in the urea cycle for the conversion of citrulline to arginosuccinate^31^. The perturbation of both mitochondria and TCA cycle could affect arginine biosynthesis as arginine level remained low after three days. We speculate that the decreased ornithine level could also suggest that ornithine was potentially being used in polyamine biosynthesis as serum and insulin can activate ornithine decarboxylase which catalyses ornithine to putrescine, a crucial precursor for polyamines^32^.

Similar to the report by Cheng et al*.*^28^, our data also indicated that glutamine was consistently upregulated across three cell lines, MDA-MB-231, A549 and H1975, and asparagine was upregulated across MDA-MB-231 and A549. Our MetPA analyses did not show any huge disparity between the pathways that were significantly impacted due to arginine starvation, suggesting that the addition of insulin did not significantly alter cellular metabolism in vitro.

Finally, we also showed that arginine deprivation by ARG induced apoptosis and not autophagy at early timepoints. Even though insulin has anti-apoptotic effects when cells are serum starved^33^, the combination of ARG and insulin did not prevent cells from undergoing apoptosis. Autophagy is a critical protective mechanism during nutrient deprivation, and play an important role in supporting tumour growth^34^. Previously Poillet-Perez et al*.* demonstrated that autophagy of host cells could lead to the release of arginine and subsequent increase in host serum arginine level in vivo^9,35^. However, we found no evidence that the cancer cells themselves underwent autophagy in vitro under ARG treatment conditions with / without insulin in our study, suggesting intracellular arginine levels would have remained low and unable to support cellular growth.

From our study we concluded that although insulin plays a role in protein metabolism, cell survival and proliferation, it does not provide cancer cells with any survival advantage in vitro under arginine starvation conditions. In vivo*,* insulin could prevent the body from breaking down muscle tissues to compensate for the depletion of arginine, thus preventing the replenishment of extracellular arginine pools. Indeed, insulin would be expected to affect multiple other pathways in vivo including amino acid uptake into cells*,* so it will be important to test the combination of ARG and insulin in this context to further our understanding of the impact insulin has on ARG-treatment strategies.

## Materials and methods

### Cell culture

The cell lines and growth media used in this research are listed in Table 1. Cell lines were kindly provided by Associate Professor Fiona Simpson (The University of Queensland), Associate Professor Andrew Brooks (The University of Queensland), and Professor Brian Gabrielli (Mater Research). Complete media consisted of growth media supplemented with 10% (v/v) heat-inactivated foetal bovine serum (FBS) (Gibco, ThermoFisher), 2 mM L-Glutamine (Gibco, ThermoFisher), and 10 mM HEPES (Gibco, ThermoFisher). All cells were grown in a humidified incubator at 37 °C with 5% CO_2_.

**Table 1:** Cell lines used in this study and relevant growth media.

Cell line	Cancer type	Growth media
SKBR3	ER^−^, PR^−^, HER2^+^ breast cancer	McCoy’s 5A
MDA-MB-453	ER^−^, PR^−^, HER2^+^ breast cancer	Roswell park memorial institute (RPMI) 1640
MCF7	ER^+^, PR^+^, HER2^−^ breast cancer	Dulbecco’s modified eagle medium (DMEM)
T47D	ER^+^, PR^+^, HER2^−^ breast cancer	RPMI 1640
MDA-MB-231	ER^−^, PR^−^, HER2^−^ triple negative breast cancer (TNBC)	DMEM
MDA-MB-468	TNBC	DMEM
A549	Non-small-cell lung cancer (NSCLC)	DMEM
HCC827	NSCLC	RPMI 1640
H1975	NSCLC	RPMI 1640
PEO1	Ovarian cancer	RPMI 1640
OVCA420	Ovarian cancer	RPMI 1640
KURAMOCHI	Ovarian cancer	RPMI 1640

### Drug and antibodies

Human recombinant ARG-1 was kindly provided by Hepius Biotech AG. Prior to use, ARG was activated with 0.5 mM MnCl_2_ at 50 °C for 10 min. 1 U/mL of ARG was calculated equivalent to 0.001 mg/mL. Recombinant human insulin was purchased from Sigma-Aldrich. Antibodies were purchased from commercial sources as follows; anti-Insulin receptor (InsR, ab131238, Abcam), anti-ARG-1 (#93,668, Cell Signalling Technology), anti-β-Tubulin (#322,600, ThermoFisher), anti-mouse IgG-horseradish peroxidase (HRP) (#7076, Cell Signalling Technology) and anti-rabbit IgG-HRP (#7074, Cell Signalling Technology).

### Cell proliferation

Cell proliferation was determined using IncuCyte® SX5 and S3 Live-Cell Analysis instruments (Satorius). Cells were seeded in a 96-well clear, flat-bottomed plate at a density of 2000 cells per well in 100 μL of media, and were allowed to attach overnight at 37 °C. Cells were treated with either MnCl_2_ only controls, 1 U/mL ARG, 10 U/mL ARG, 1 nM insulin, 10 nM insulin or combinations of ARG and insulin as indicated in figure legends. Cells were treated for 9 days before being changed into complete media. Cell proliferation was subsequently assessed over the following 8 days. Images were taken at 2-h intervals for the full 17 days. Cell proliferation was determined by area of cell confluency using the IncuCyte® analysis software.

### Apoptosis assay

Cells were treated with the same treatment conditions mentioned above for 3 days. Cells were harvested through trypsinisation and washed with PBS before staining with Annexin V-FITC (#640,906, Biolegend) for 15 min at a dilution of 1:20 in Annexin V binding buffer (#422,201, Biolegend). Cells were washed twice in Annexin V binding buffer and stained with 7-AAD (BD Biosciences) at a dilution of 1:20 before analysing with BD LSRFortessa™ X-20 (BD Biosciences). All staining was performed at room temperature and data was analysed in FlowJo software (BD Biosciences).

### Autophagy assay

Autophagy was detected using an Autophagy Detection Kit (#ab139484, abcam) performed according to the manufacturer’s instructions. Briefly, cells were seeded at 10,000 cells/well in 100 μL of media and were allowed to attach overnight at 37 °C. Cells were treated with the same treatment conditions mentioned above for 24 h before staining with both autophagy detection stain and nuclear stain included in the kit for 30 min at 37 °C in the dark. Samples were then washed twice with the assay buffer provided. Data was acquired immediately with a fluorescence microplate reader (CLARIOstar Plus, BMG Labtech) using FITC and DAPI filters.

### Amino acid analysis

Cells were seeded in a 10-cm dish and were allowed to attach overnight at 37 °C. Cells were treated with MnCl_2_, 1 nM insulin, 1 U/mL ARG ± 1 nM insulin, and 10 U/mL ARG ± 1 nM insulin for 3 days. Whole cell lysates were harvested by lysing cells on ice with Radioimmunoprecipitation Assay (RIPA) buffer supplemented with phosphatases and proteinase inhibitors (Roche, Merck KGaA). Cell lysates were sonicated and centrifuged at top speed for 15 min and supernatants collected. Supernatants were then immediately stored at − 80 °C before sending to Queensland Metabolomics and Proteomics (Q-MAP, The University of Queensland, Australia) for amino acid analysis by high performance liquid chromatography (HPLC).

### Metabolomic data processing and statistical analysis

Univariate statistical analyses were performed on metabolomic data obtained from amino acid analysis mentioned above using the web-based software, MetaboAnalyst 5.0^36,37^. Before performing any analysis, data was subjected to log transformation and data scaling to mean centring. Univariate analyses performed include fold-change analyses and T-tests, and volcano plots were generated from the results. For the volcano plots, fold-change threshold was set at 1.5, with direction of comparison as Treatment/ Control, and *p*-value threshold was set as 0.1 false discovery rate (FDR). Metabolomic Pathway Analysis (MetPA) was also performed using MetaboAnalyst 5.0. Metabolomic data detected in samples were submitted into MetPA as KEGG ID, and verified using KEGG database. Global test was selected as the enrichment method, and relative-betweenness centrality was selected for topology analysis. A Homo sapiens (KEGG) pathway was used for the pathway analysis.

### Western blot

Cells were seeded in a 10-cm dish and were allowed to attach overnight at 37 °C. Cells were treated with either MnCl2 control, 1 U/mL ARG, 1 nM insulin or a combination of 1 U/mL ARG and 1 nM insulin for 24 h. Whole cell protein lysates were harvested by lysing cells on ice with RIPA buffer supplemented with phosphatases and proteinase inhibitors (Roche, Merck KGaA). Cell lysates were sonicated and centrifuged at top speed for 15 min and supernatant were collected. Protein concentrations were subsequently determined using Pierce BCA Assay Kit (ThermoFisher) according to the manufacturer’s protocol. Equal amount of protein samples were separated on 10% SDS-PAGE and transferred onto a nitrocellulose membrane (GE Healthcare) electrophoretically. Membranes were incubated in blocking buffer (5% w/v bovine serum albumin (BSA, Sigma-Aldrich) in TBS-T (20 mM Tris–HCl, pH7.4/137 mM NaCl/ 0.1% (v/v) Tween-20)) for 1 h at room temperature. Membranes were then incubated with primary antibodies at a dilution of 1:1000 in blocking buffer overnight at 4 °C with shaking. Membranes were washed in TBS-T before incubating with appropriate HRP-conjugated secondary antibodies at a dilution of 1:4000 in blocking buffer for 1 h at room temperature with shaking. β-Tubulin was used as loading control and membranes were incubated with anti- β-Tubulin at a dilution of 1:10,000 in blocking buffer for 1 h at room temperature. Membranes were then visualised using enhanced chemiluminescence (ECL) on ChemiDoc XRS+ (Bio-Rad). The relative expression of specific protein was determined through quantification of scanned images using ImageJ.

### Statistical analysis

Data obtained from apoptosis and autophagy assays, and Western blot quantification were plotted using GraphPad Prism (Version 9.4.1) for Windows, GraphPad Software, San Diego, California USA, www.graphpad.com. Statistical analysis between different treatment groups was performed using One-way ANOVA followed by Tukey’s multiple comparisons test.

### Supplementary Information

Supplementary Information.

## Data Availability

All data generated or analysed during this study are included in this published article (and its Supplementary Information files).

## References

[1] Al-Koussa H, El Mais N, Maalouf H, Abi-Habib R, El-Sibai M (2020). Arginine deprivation: A potential therapeutic for cancer cell metastasis? A review. Cancer Cell Int..

[2] Feun L, You M, Wu CJ, Kuo MT, Wangpaichitr M, Spector S (2008). Arginine deprivation as a targeted therapy for cancer. Curr. Pharm. Des..

[3] Wu G, Bazer FW, Davis TA, Kim SW, Li P, Marc Rhoads J (2009). Arginine metabolism and nutrition in growth, health and disease. Amino Acids.

[4] Wells JW, Evans CH, Scott MC, Rutgen BC, O'Brien TD, Modiano JF (2013). Arginase treatment prevents the recovery of canine lymphoma and osteosarcoma cells resistant to the toxic effects of prolonged arginine deprivation. PLoS One.

[5] Chan SL, Cheng PNM, Liu AM, Chan LL, Li L, Chu CM (2021). A phase II clinical study on the efficacy and predictive biomarker of pegylated recombinant arginase on hepatocellular carcinoma. Invest. New Drugs.

[6] De Santo C, Cheng P, Beggs A, Egan S, Bessudo A, Mussai F (2018). Metabolic therapy with PEG-arginase induces a sustained complete remission in immunotherapy-resistant melanoma. J. Hematol. Oncol..

[7] Cheng PNM, Liu AM, Bessudo A, Mussai F (2021). Safety, PK/PD and preliminary anti-tumor activities of pegylated recombinant human arginase 1 (BCT-100) in patients with advanced arginine auxotrophic tumors. Invest. New Drugs.

[8] Chan PY, Phillips MM, Ellis S, Johnston A, Feng X, Arora A (2022). A Phase 1 study of ADI-PEG20 (pegargiminase) combined with cisplatin and pemetrexed in ASS1-negative metastatic uveal melanoma. Pigment Cell Melanoma Res..

[9] Poillet-Perez L, Xie X, Zhan L, Yang Y, Sharp DW, Hu ZS (2018). Autophagy maintains tumour growth through circulating arginine. Nature.

[10] Pisters PW, Cersosimo E, Rogatko A, Brennan MF (1992). Insulin action on glucose and branched-chain amino acid metabolism in cancer cachexia: Differential effects of insulin. Surgery.

[11] Cheng PN, Leung YC, Lo WH, Tsui SM, Lam KC (2005). Remission of hepatocellular carcinoma with arginine depletion induced by systemic release of endogenous hepatic arginase due to transhepatic arterial embolisation, augmented by high-dose insulin: Arginase as a potential drug candidate for hepatocellular carcinoma. Cancer Lett..

[12] Dabir S, Dabir P, Somvanshi B (2006). The kinetics of inhibition of Vigna catjang cotyledon and buffalo liver arginase by L-proline and branched-chain amino acids. J. Enzyme Inhib Med. Chem..

[13] Vigneri R, Sciacca L, Vigneri P (2020). Rethinking the relationship between Insulin and Cancer. Trends Endocrinol. Metab..

[14] Dillon BJ, Prieto VG, Curley SA, Ensor CM, Holtsberg FW, Bomalaski JS (2004). Incidence and distribution of argininosuccinate synthetase deficiency in human cancers: A method for identifying cancers sensitive to arginine deprivation. Cancer.

[15] Lam TL, Wong GK, Chow HY, Chong HC, Chow TL, Kwok SY (2011). Recombinant human arginase inhibits the in vitro and in vivo proliferation of human melanoma by inducing cell cycle arrest and apoptosis. Pigment Cell Melanoma Res..

[16] Lamb J, Wheatley DN (2000). Single amino acid (arginine) deprivation induces G1 arrest associated with inhibition of cdk4 expression in cultured human diploid fibroblasts. Exp. Cell Res..

[17] Lotspeich WD (1949). The role of insulin in the metabolism of amino acids. J. Biol. Chem..

[18] Shin AC, Fasshauer M, Filatova N, Grundell LA, Zielinski E, Zhou JY (2014). Brain insulin lowers circulating BCAA levels by inducing hepatic BCAA catabolism. Cell Metab..

[19] Frendo-Cumbo S, Tokarz VL, Bilan PJ, Brumell JH, Klip A (2021). Communication between autophagy and insulin action: At the Crux of insulin action-insulin resistance?. Front. Cell Dev. Biol..

[20] Gallagher EJ, LeRoith D (2010). The proliferating role of insulin and insulin-like growth factors in cancer. Trends Endocrinol. Metab..

[21] Lu CC, Chu PY, Hsia SM, Wu CH, Tung YT, Yen GC (2017). Insulin induction instigates cell proliferation and metastasis in human colorectal cancer cells. Int. J. Oncol..

[22] Rose DP, Vona-Davis L (2012). The cellular and molecular mechanisms by which insulin influences breast cancer risk and progression. Endocr. Relat. Cancer.

[23] Chen CL, Hsu SC, Ann DK, Yen Y, Kung HJ (2021). Arginine signaling and cancer metabolism. Cancers.

[24] Chantranupong L, Scaria SM, Saxton RA, Gygi MP, Shen K, Wyant GA (2016). The CASTOR proteins are arginine sensors for the mTORC1 pathway. Cell.

[25] Saxton RA, Chantranupong L, Knockenhauer KE, Schwartz TU, Sabatini DM (2016). Mechanism of arginine sensing by CASTOR1 upstream of mTORC1. Nature.

[26] Wang S, Tsun ZY, Wolfson RL, Shen K, Wyant GA, Plovanich ME (2015). Metabolism. Lysosomal amino acid transporter SLC38A9 signals arginine sufficiency to mTORC1. Science.

[27] Saxton RA, Sabatini DM (2017). mTOR signaling in growth, metabolism, and disease. Cell.

[28] Cheng CT, Qi Y, Wang YC, Chi KK, Chung Y, Ouyang C (2018). Arginine starvation kills tumor cells through aspartate exhaustion and mitochondrial dysfunction. Commun. Biol..

[29] Hu J, Locasale JW, Bielas JH, O'Sullivan J, Sheahan K, Cantley LC (2013). Heterogeneity of tumor-induced gene expression changes in the human metabolic network. Nat. Biotechnol..

[30] Gaude E, Frezza C (2016). Tissue-specific and convergent metabolic transformation of cancer correlates with metastatic potential and patient survival. Nat. Commun..

[31] Keshet R, Szlosarek P, Carracedo A, Erez A (2018). Rewiring urea cycle metabolism in cancer to support anabolism. Nat. Rev. Cancer.

[32] Hogan B, Shields R, Curtis D (1974). Effect of cyclic nucleotides on the induction of ornithine decarboxylase in BHK cells by serum and insulin. Cell..

[33] Kang S, Song J, Kang H, Kim S, Lee Y, Park D (2003). Insulin can block apoptosis by decreasing oxidative stress via phosphatidylinositol 3-kinase- and extracellular signal-regulated protein kinase-dependent signaling pathways in HepG2 cells. Eur. J. Endocrinol..

[34] Kimmelman AC, White E (2017). Autophagy and tumor metabolism. Cell Metab..

[35] Lei S, Fei R, Lei L (2019). Autophagy elicits a novel and prospect strategy to starve arginine-dependent tumors. Hepatobiliary Surg. Nutr..

[36] Pang Z, Chong J, Zhou G, van Lima Morais DA, Chang L, Barrette M (2021). MetaboAnalyst 5.0: Narrowing the gap between raw spectra and functional insights. Nucleic Acids Res..

[37] Xia J, Wishart DS (2011). Metabolomic data processing, analysis, and interpretation using MetaboAnalyst. Curr. Protoc. Bioinforma..

