# Supplementary material for: Arginase-induced cell death pathways and metabolic changes in cancer cells are not altered by insulin
Source: Sci Rep. 2024 Feb 19;14:4112. doi: 10.1038/s41598-024-54520-z (PMC10876525; doi:10.1038/s41598-024-54520-z)
Supplement: Supplementary file 1 — Supplementary Information. [file 41598_2024_54520_MOESM1_ESM.pdf]

Supplementary Figures

Supplementary Figure S1. Quantification of western blot presented in Figure 1.

Supplementary Figure S2. Raw images of Western blot from Main Figure 1A. **A.** Chemiluminescence detection of insulin receptor in breast cancer cell lines. **B.** Colourimetric detection of protein ladder. Protein standards were marked with a ballpoint pen accordingly right after transfer due to ladder fading after multiple wash steps with 1x TBS-T. **C.** Chemiluminescence detection of β-Tubulin. **D.** Colourimetric detection of protein ladder, marked accordingly. Molecular weights of the protein ladder are indicated in Figure **B**.

Supplementary Figure S3. Raw images of Western blot from Main Figure 1B. **A.** Chemiluminescence detection of insulin receptor in lung cancer cell lines. **B.** Colourimetric detection of protein ladder. Protein standards were marked with a ballpoint pen accordingly right after transfer due to ladder fading after multiple wash steps with 1x TBS-T. **C.** Chemiluminescence detection of β-Tubulin. **D.** Colourimetric detection of protein ladder, marked accordingly. Images were cropped to only show the blot in the figure as multiple blots were developed together. Molecular weights of the protein ladder are indicated in **Supplementary Figure S2B**.

Supplementary Figure S4. Raw images of Western blot from Main Figure 1C. **A.** Chemiluminescence detection of insulin receptor in ovarian cancer cell lines. **B.** Colourimetric detection of protein ladder. Protein standards were marked with a ballpoint pen accordingly right after transfer due to ladder fading after multiple wash steps with 1x TBS-T. **C.** Chemiluminescence detection of β-Tubulin. **D.** Colourimetric detection of protein ladder, marked accordingly. Images were cropped to only show the blot in the figure as multiple blots were developed together. Molecular weights of the protein ladder are indicated in **Supplementary Figure S2B**.

Supplementary Figure S5. Raw images of Western blot from Main Figure 1D. **A.** Chemiluminescence detection of arginase (ARG) in MDA-MB-453 cells. **B.** Colourimetric detection of protein ladder. Protein standards were marked with a ballpoint pen accordingly right after transfer due to ladder fading after multiple wash steps with 1x TBS-T. **C.** Chemiluminescence detection of β-Tubulin. **D.** Colourimetric detection of protein ladder, marked accordingly. Images were cropped to only show the blot in the figure as multiple blots were developed together. Molecular weights of the protein ladder are indicated in **Supplementary Figure S2B**.

Supplementary Figure S6. Raw images of Western blot from Main Figure 1E. **A.** Chemiluminescence detection of ARG in MCF7 cells. **B.** Colourimetric detection of protein ladder. Protein standards were marked with a ballpoint pen accordingly right after transfer due to ladder fading after multiple wash steps with 1x TBS-T. **C.** Chemiluminescence detection of β-Tubulin. **D.** Colourimetric detection of protein ladder, marked accordingly. Molecular weights of the protein ladder are indicated in **Supplementary Figure S2B**.

Supplementary Figure S7. Raw images of Western blot from Main Figure 1F. **A.** Chemiluminescence detection of ARG in MDA-MB-231 cells. **B.** Colourimetric detection of protein ladder. Protein standards were marked with a ballpoint pen accordingly right after transfer due to ladder fading after multiple wash steps with 1x TBS-T. **C.** Chemiluminescence

detection of β-Tubulin. **D.** Colourimetric detection of protein ladder, marked accordingly. Images from C and D were cropped to only show the blot in the figure as multiple blots were developed together. Molecular weights of the protein ladder are indicated in **Supplementary Figure S2B**.

Supplementary Figure S8. Raw images of Western blot from Main Figure 1G. **A.** Chemiluminescence detection of ARG in A549 cells. **B.** Colourimetric detection of protein ladder. Protein standards were marked with a ballpoint pen accordingly right after transfer due to ladder fading after multiple wash steps with 1x TBS-T. **C.** Chemiluminescence detection of β-Tubulin. **D.** Colourimetric detection of protein ladder, marked accordingly. Images were cropped to only show the blot in the figure as multiple blots were developed together. Molecular weights of the protein ladder are indicated in **Supplementary Figure S2B**.

Supplementary Figure S9. Raw images of Western blot from Main Figure 1H. **A.** Chemiluminescence detection of ARG in HCC827 cells. **B.** Colourimetric detection of protein ladder. Protein standards were marked with a ballpoint pen accordingly right after transfer due to ladder fading after multiple wash steps with 1x TBS-T. **C.** Chemiluminescence detection of β-Tubulin. **D.** Colourimetric detection of protein ladder, marked accordingly. Images were cropped to only show the blot in the figure as multiple blots were developed together. Molecular weights of the protein ladder are indicated in **Supplementary Figure S2B**.

Supplementary Figure S10. Raw images of Western blot from Main Figure 1I. **A.** Chemiluminescence detection of ARG in H1975 cells. **B.** Colourimetric detection of protein ladder. Protein standards were marked with a ballpoint pen accordingly right after transfer due to ladder fading after multiple wash steps with 1x TBS-T. **C.** Chemiluminescence detection of β-Tubulin. **D.** Colourimetric detection of protein ladder, marked accordingly. Images were cropped to only show the blot in the figure as multiple blots were developed together. Molecular weights of the protein ladder are indicated in **Supplementary Figure S2B**.

Supplementary Figure S11. Raw images of Western blot from Main Figure 1J. **A.** Chemiluminescence detection of ARG in PEO1 cells. **B.** Colourimetric detection of protein ladder. Protein standards were marked with a ballpoint pen accordingly right after transfer due to ladder fading after multiple wash steps with 1x TBS-T. **C.** Chemiluminescence detection of β-Tubulin. **D.** Colourimetric detection of protein ladder, marked accordingly. Images were cropped to only show the blot in the figure as multiple blots were developed together. Molecular weights of the protein ladder are indicated in **Supplementary Figure S2B**.

Supplementary Figure S12. Raw images of Western blot from Main Figure 1K. **A.** Chemiluminescence detection of ARG in OVCA420 cells. **B.** Colourimetric detection of protein ladder. Protein standards were marked with a ballpoint pen accordingly right after transfer due to ladder fading after multiple wash steps with 1x TBS-T. **C.** Chemiluminescence detection of β-Tubulin. **D.** Colourimetric detection of protein ladder, marked accordingly. Images were cropped to only show the blot in the figure as multiple blots were developed together. Molecular weights of the protein ladder are indicated in **Supplementary Figure S2B**.

Supplementary Figure S13. Raw images of Western blot from Main Figure 1J. **A.** Chemiluminescence detection of ARG in KURAMOCHI cells. **B.** Colourimetric detection of protein ladder. Protein standards were marked with a ballpoint pen accordingly right after transfer due to ladder fading after multiple wash steps with 1x TBS-T. **C.** Chemiluminescence detection of β-Tubulin. **D.** Colourimetric detection of protein ladder, marked accordingly.

Images were cropped to only show the blot in the figure as multiple blots were developed together. Molecular weights of the protein ladder are indicated in **Supplementary Figure S2B**.

Supplementary Figure S14. Amino acid analysis of arginine, ornithine and citrulline in cancer cell lysates. (A – C) MDA-MB-231 cell lysates, (D – F) A549 cell lysates, (G – I) H1975 cell lysates, and (J – L) KURAMOCHI cell lysates. Data presented as mean ± S.D. One-way ANOVA followed by Tukey's multiple comparisons test was used to determine *p*-values; ns $p > 0.05$, * $p \leq 0.05$, ** $p \leq 0.01$, **** $p \leq 0.0001$.

Supplementary Figure S15. Pathway analysis of differentially expressed metabolites in cancer cell lysates. The data is presented as pathway impact vs. *p*-value. The size and colour of each dot correlates with its pathway impact and *p*-value, respectively. Left: Cells treated with 1 U/mL ARG; Right: Cells treated with 10 U/mL ARG. (A – B) MDA-MB-231 cell lysates, (C – D) A549 cell lysates, (E – F) H1975 cell lysates, and (G – H) KURAMOCHI cell lysates.

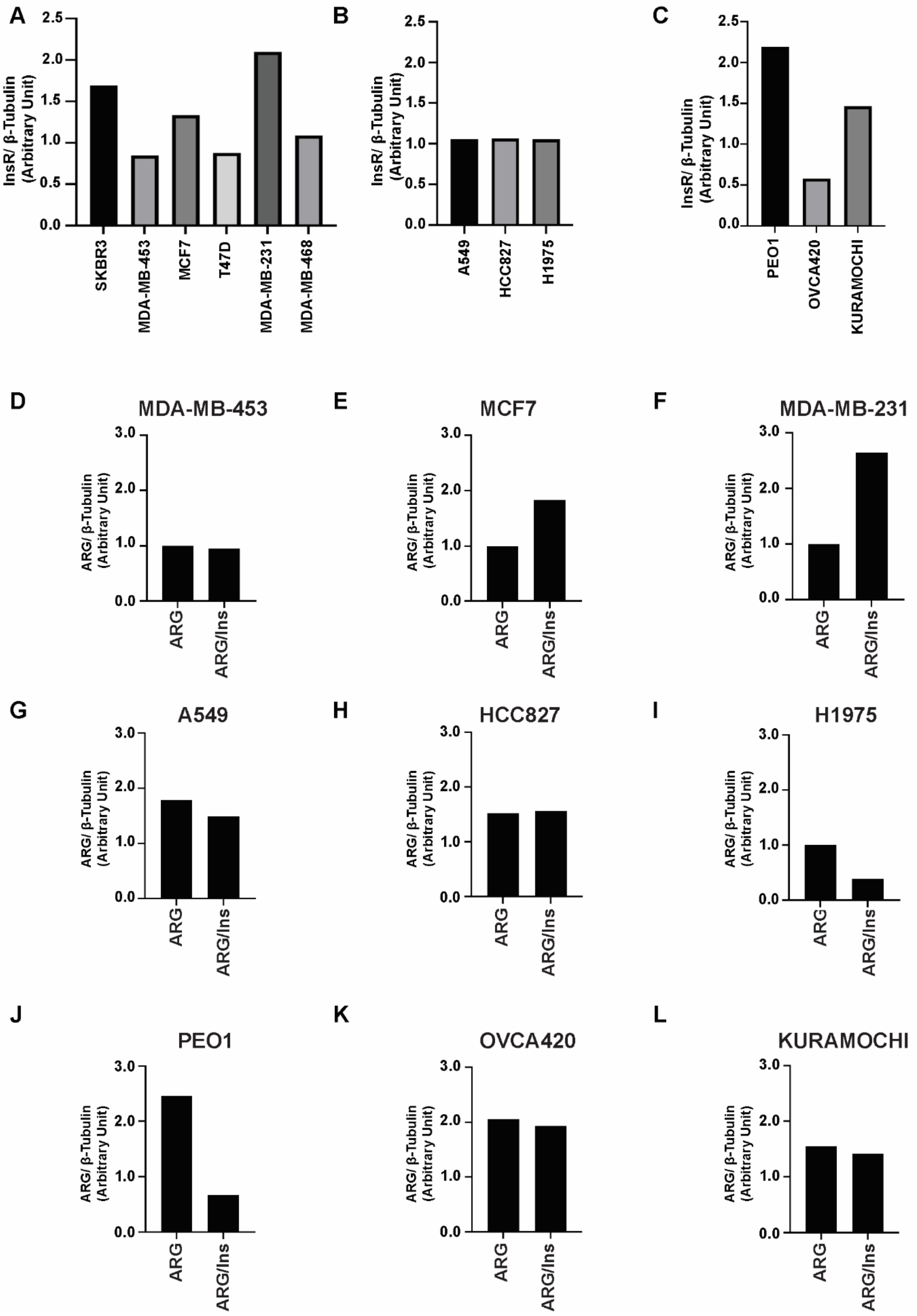

Supplementary Figure S1

A.

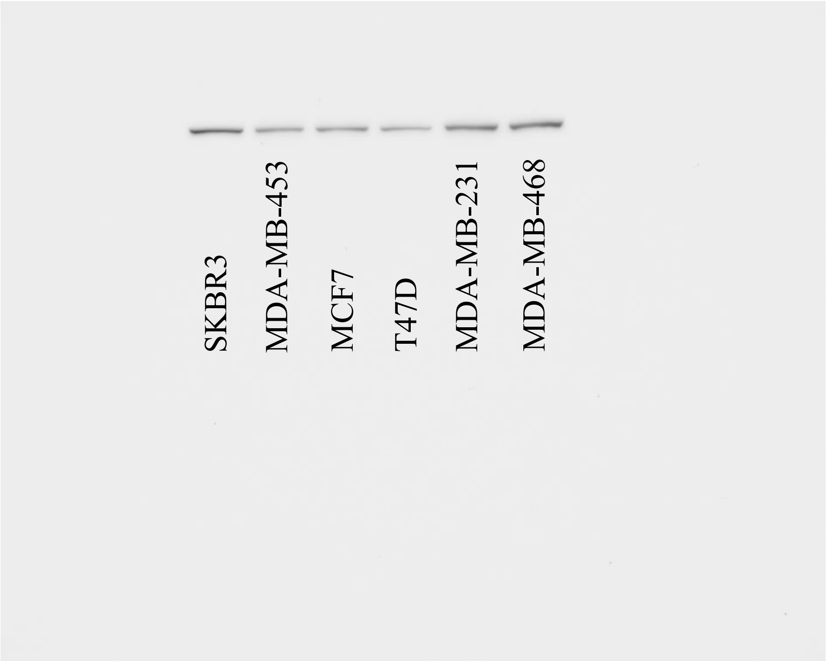

B.

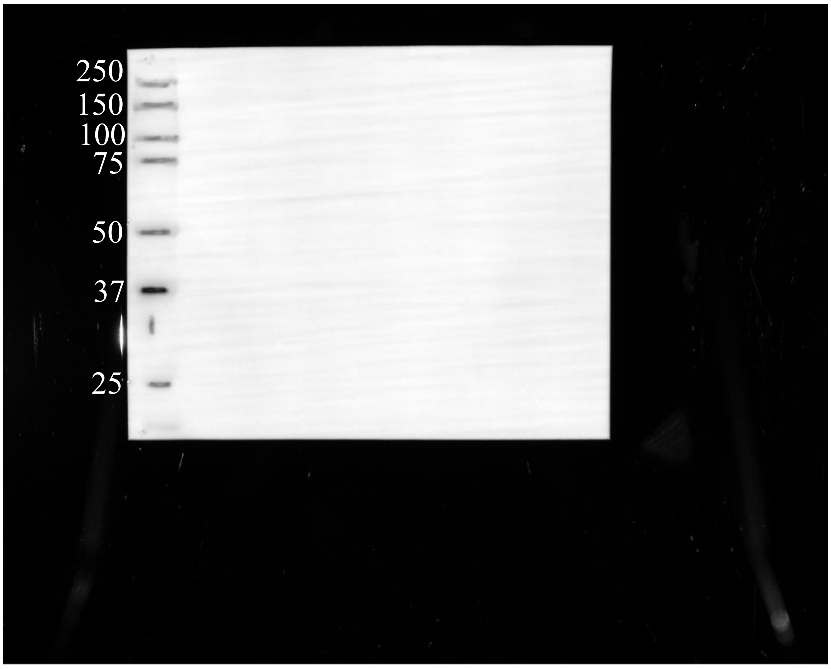

Supplementary Figure S2

C.

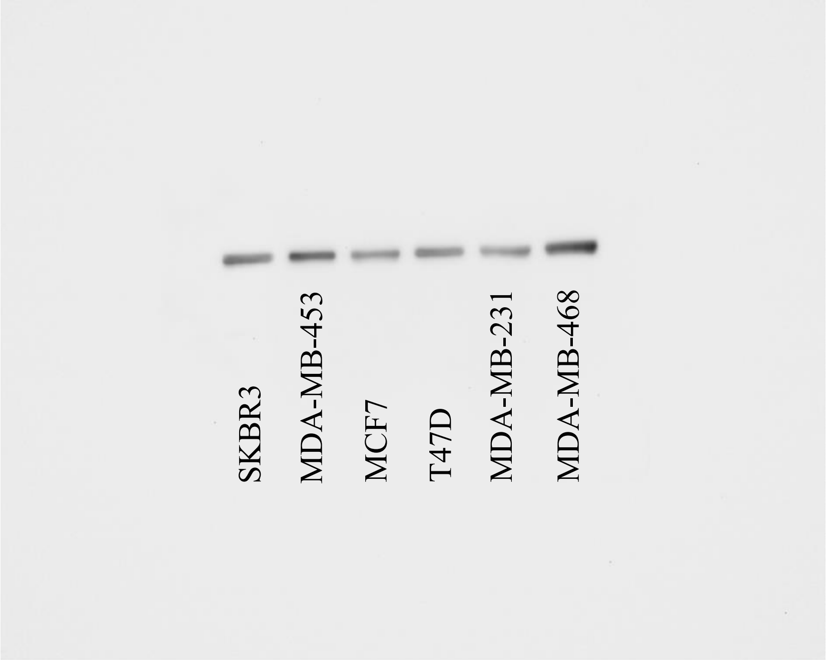

D.

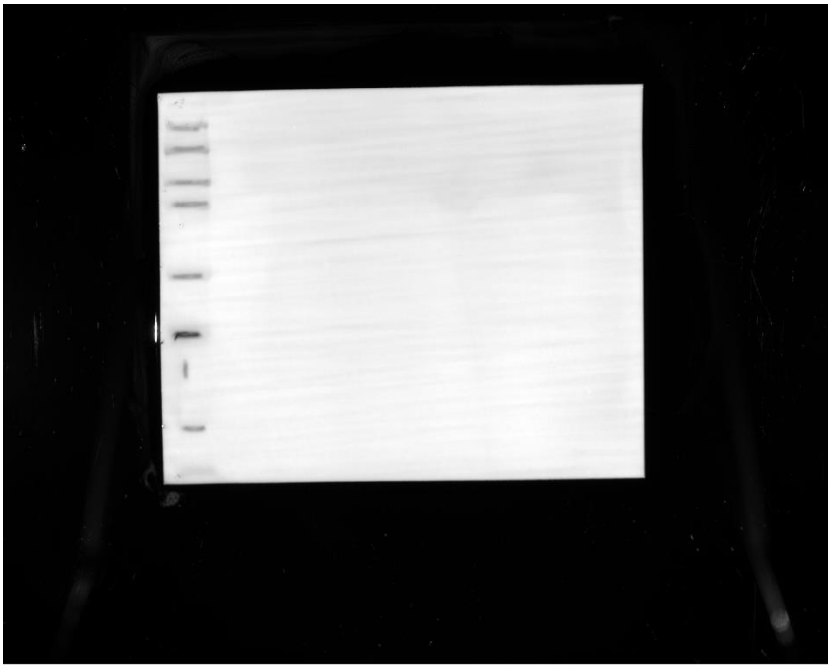

Supplementary Figure S2 continued.

A.

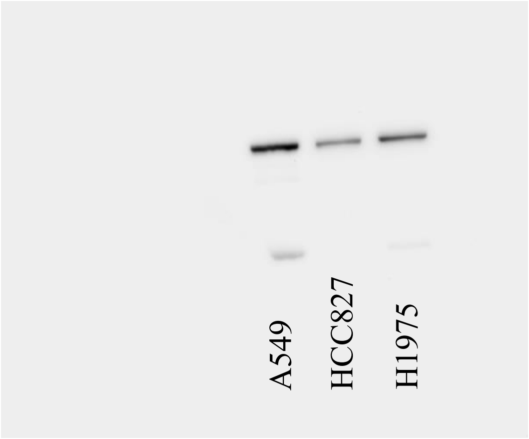

B.

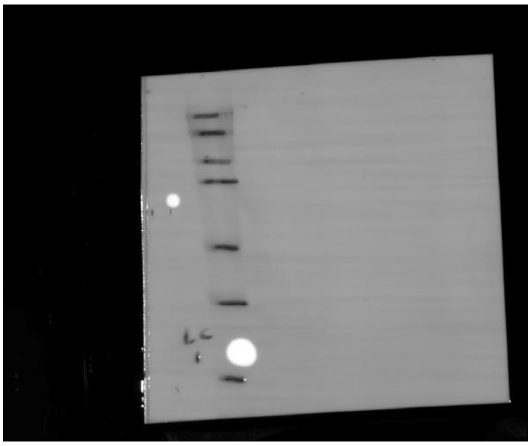

Supplementary Figure S3.

C.

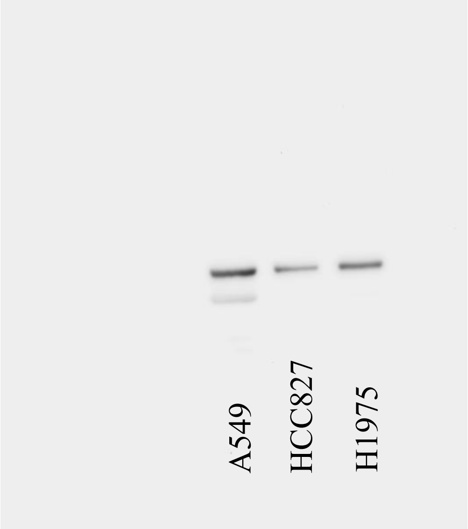

D.

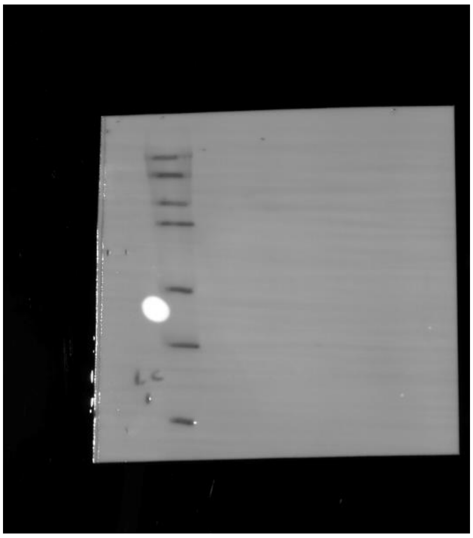

Supplementary Figure S3 continued.

A.

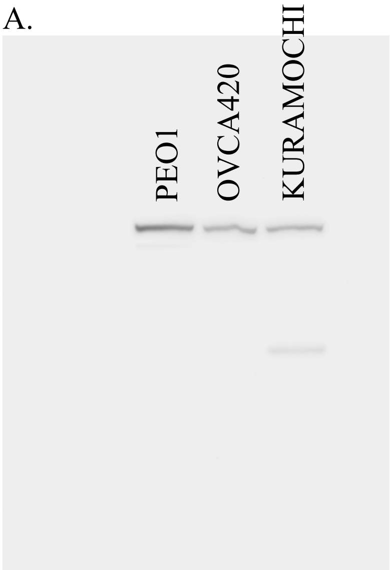

B.

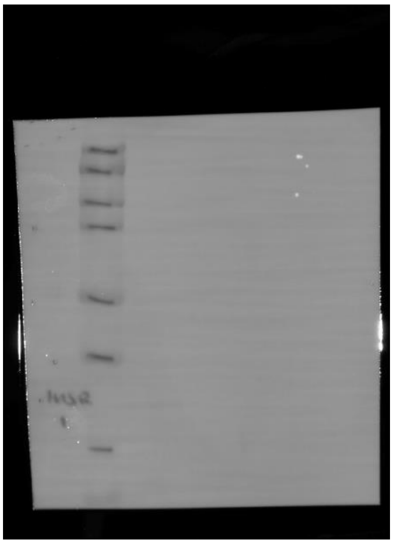

Supplementary Figure S4.

C.

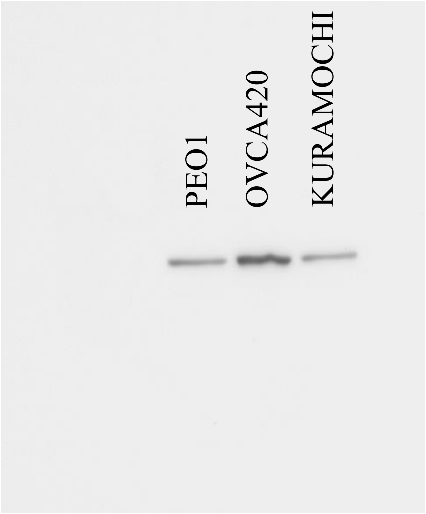

D.

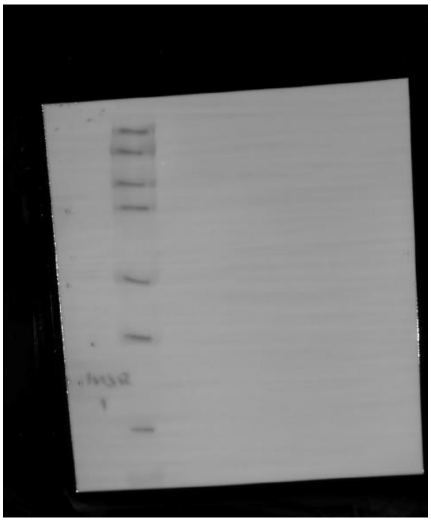

Supplementary Figure S4 continued.

A.

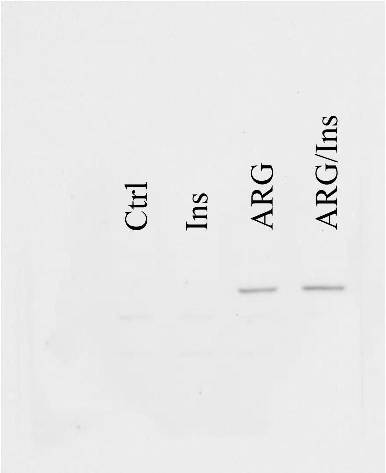

B.

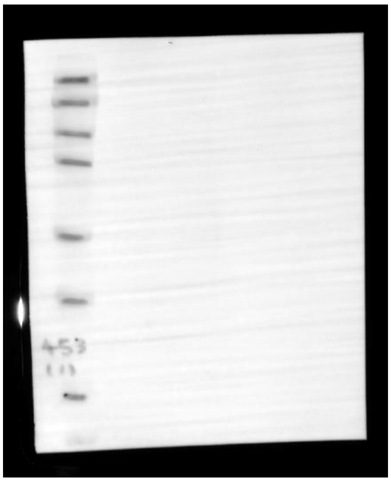

Supplementary Figure S5

C.

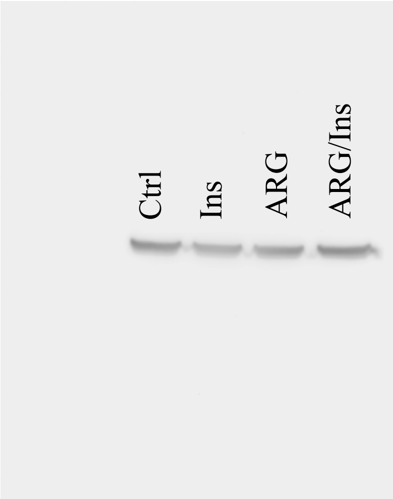

D.

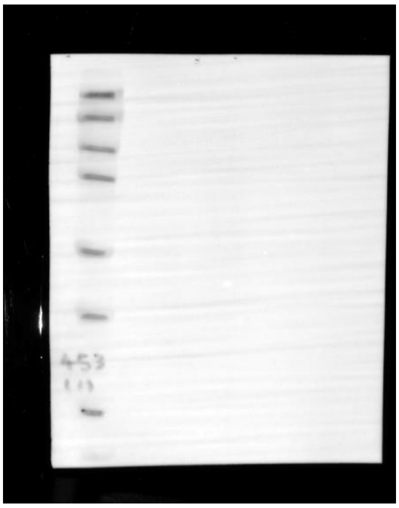

Supplementary Figure S5 continued

A.

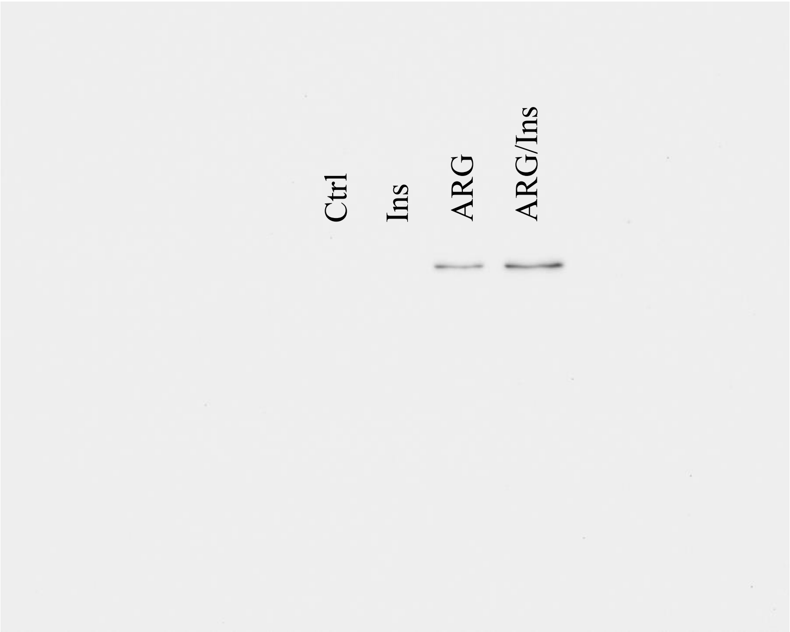

B.

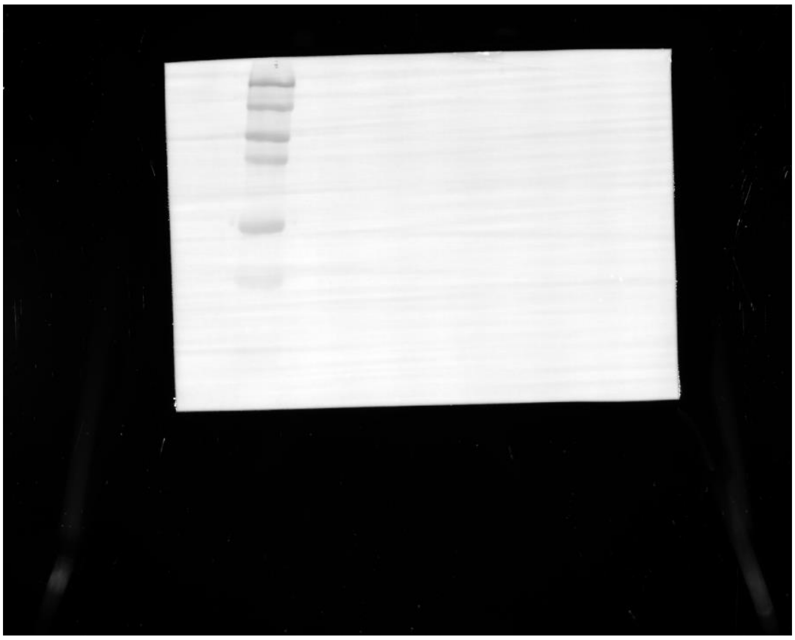

Supplementary Figure S6.

C.

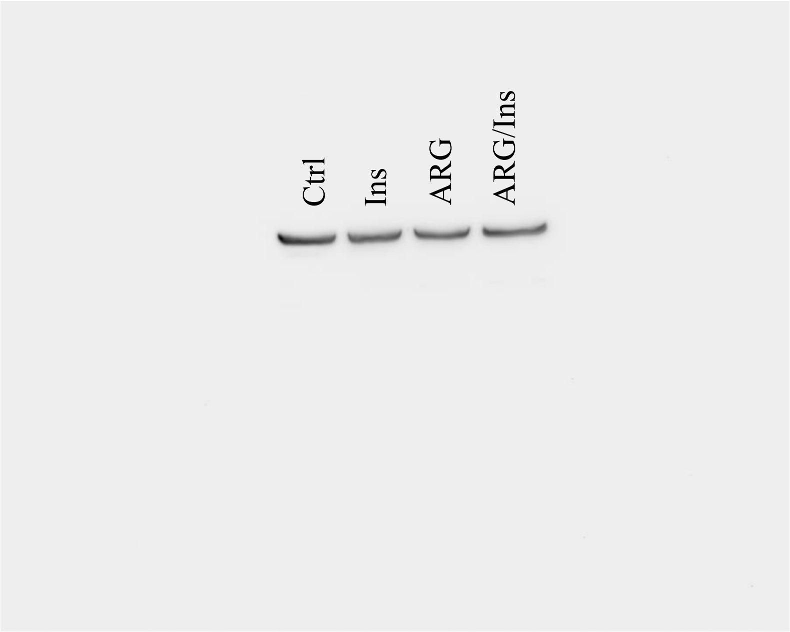

D.

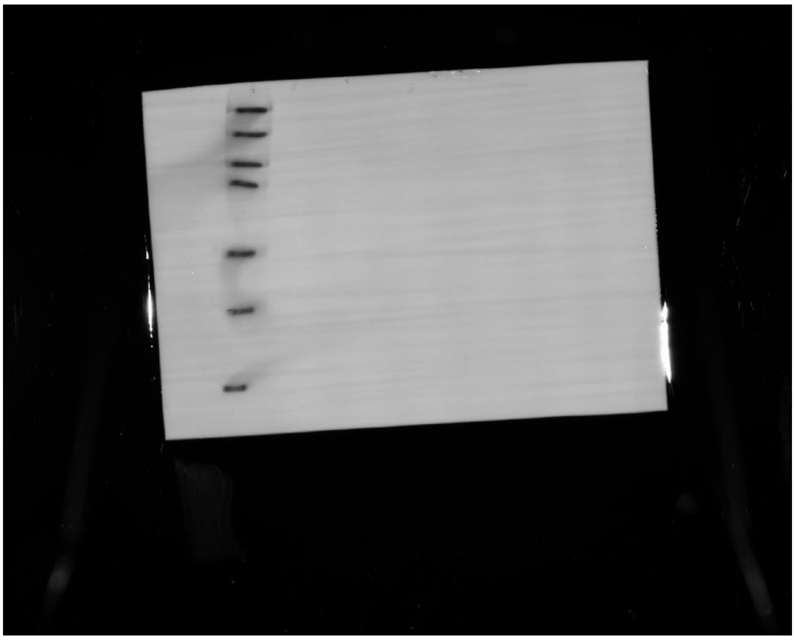

Supplementary Figure S6 continued.

A.

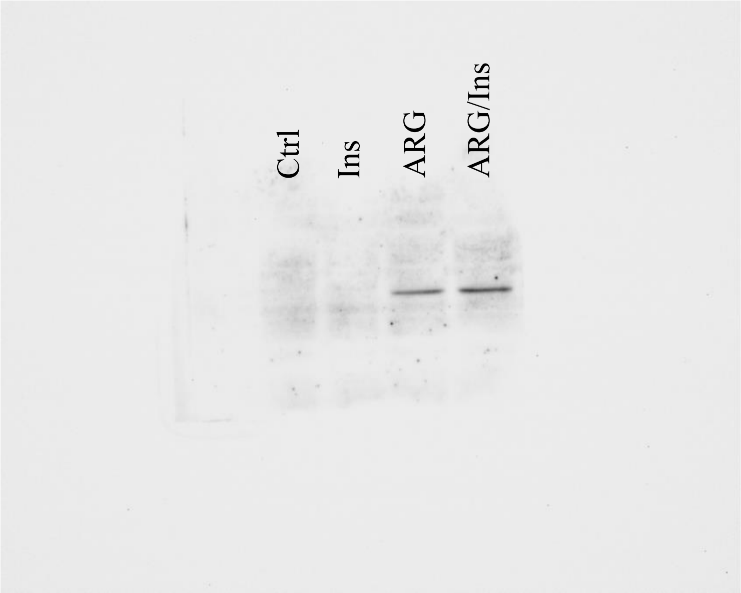

B.

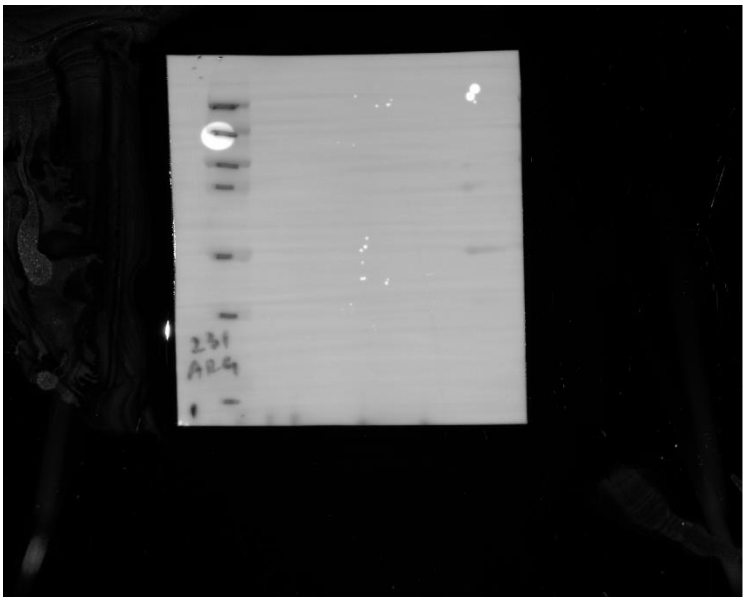

Supplementary Figure S7.

C.

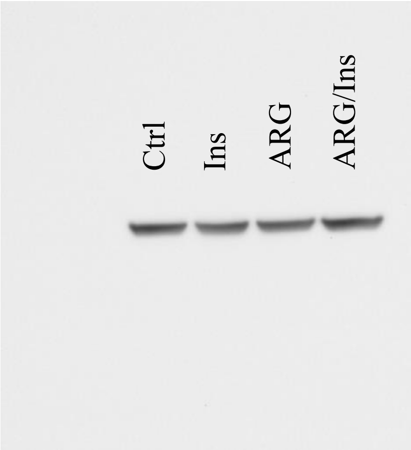

D.

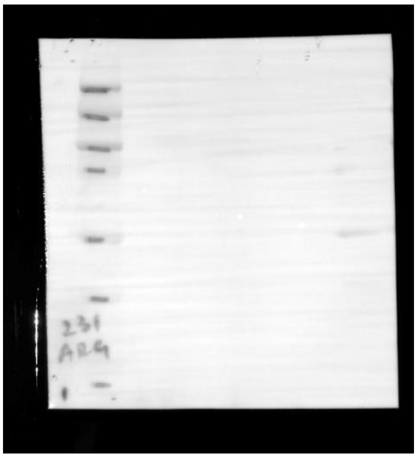

Supplementary Figure S7 continued.

A.

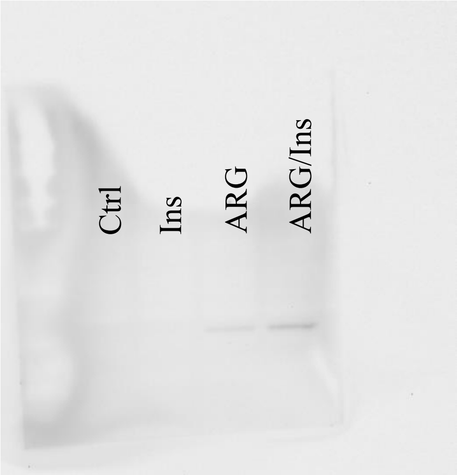

B.

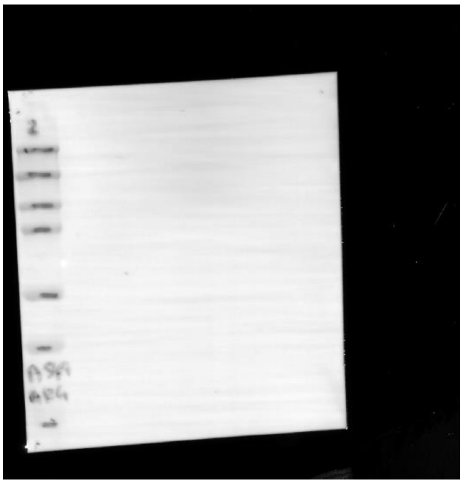

Supplementary Figure S8.

C.

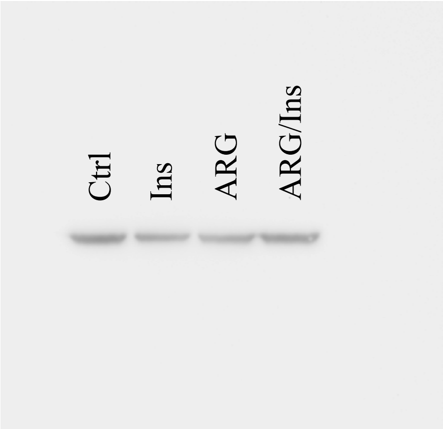

D.

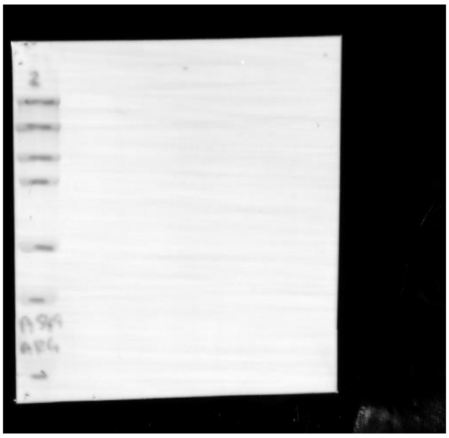

Supplementary Figure S8 continued.

A.

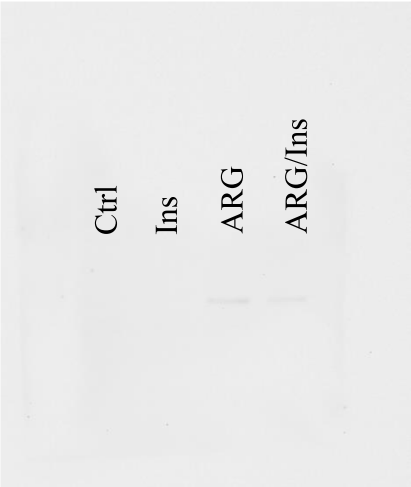

B.

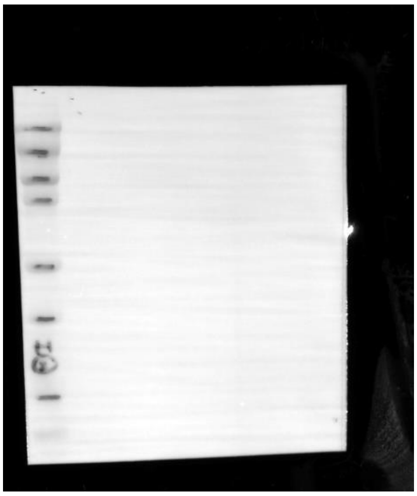

Supplementary Figure S9

C.

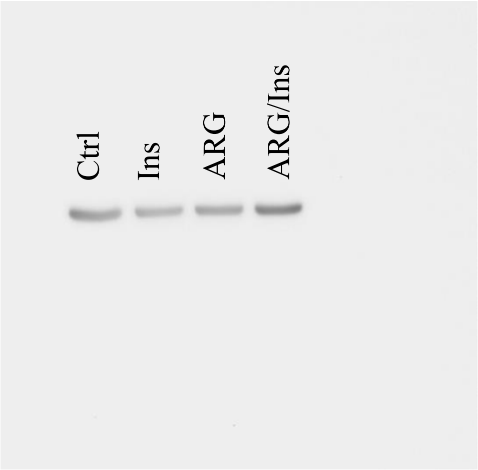

D.

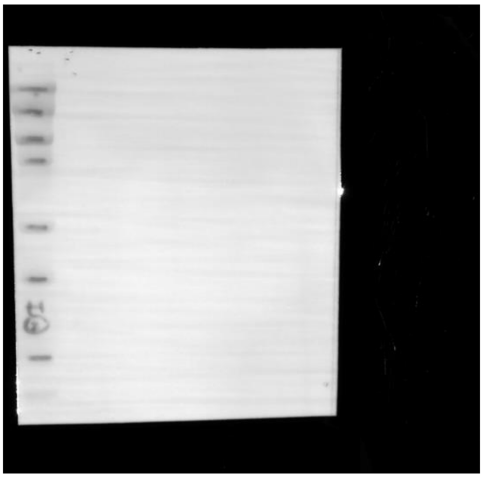

Supplementary Figure S9 continued.

A.

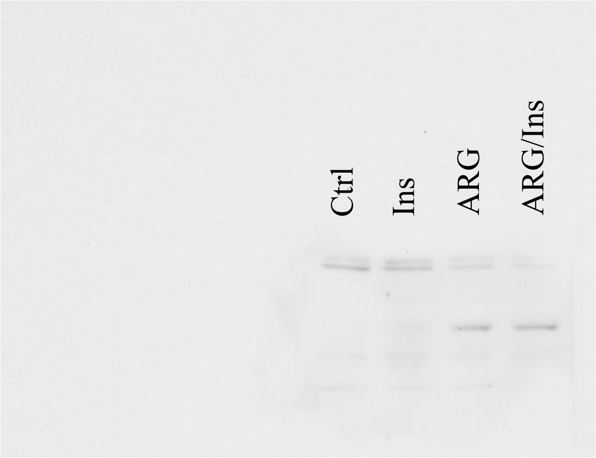

B.

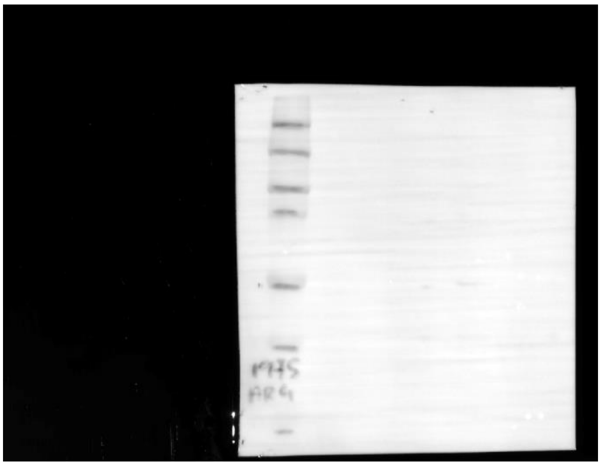

Supplementary Figure S10.

C.

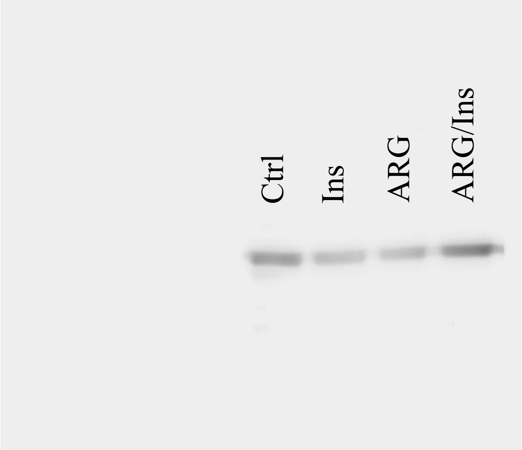

D.

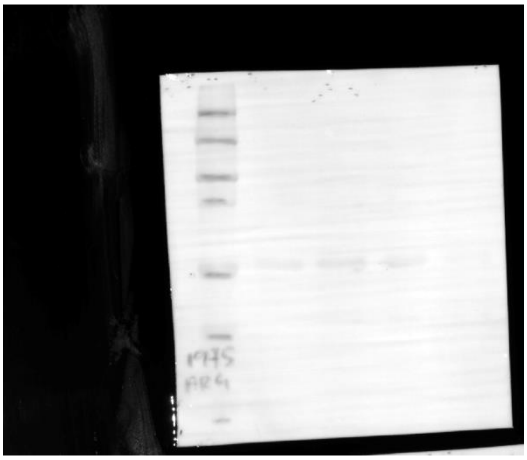

Supplementary Figure S10 continued.

A.

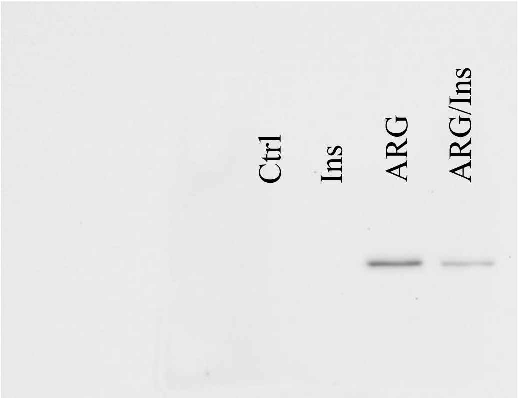

B.

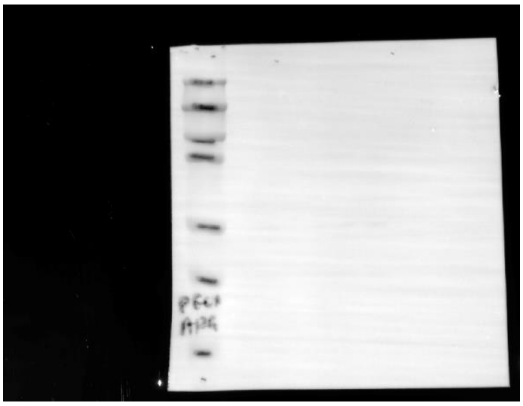

Supplementary Figure S11.

C.

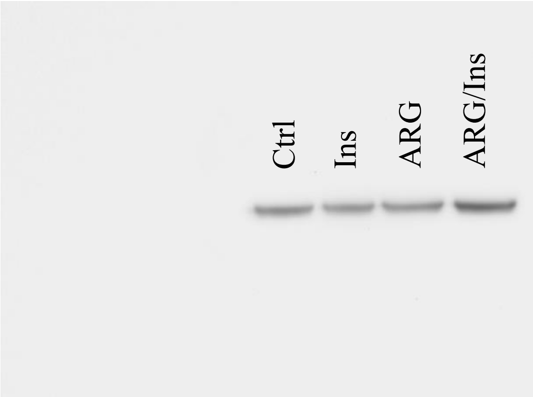

D.

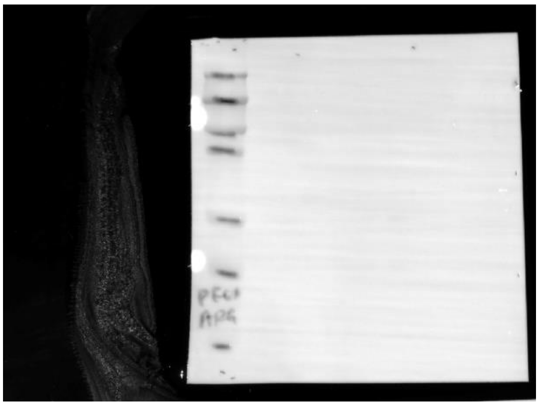

Supplementary Figure S11 continued.

A.

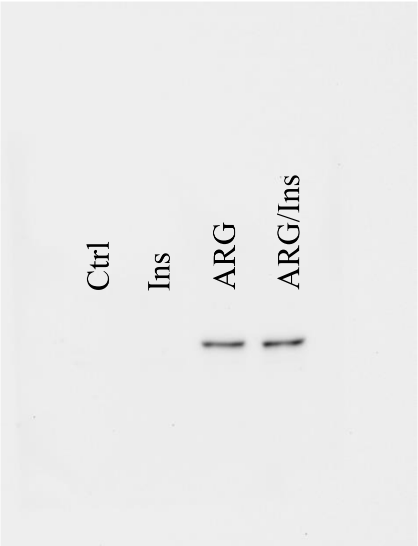

B.

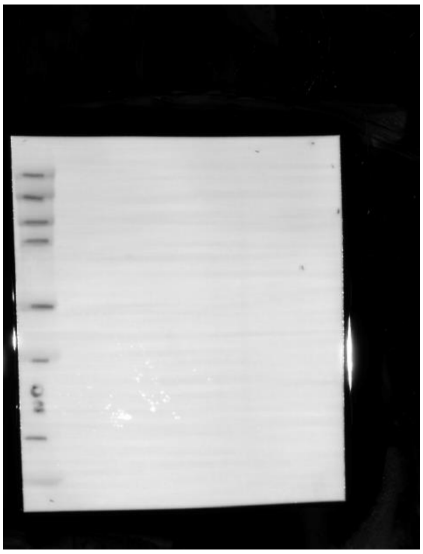

Supplementary Figure S12

C.

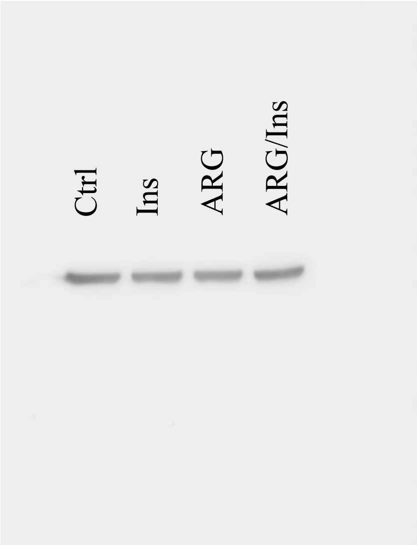

D.

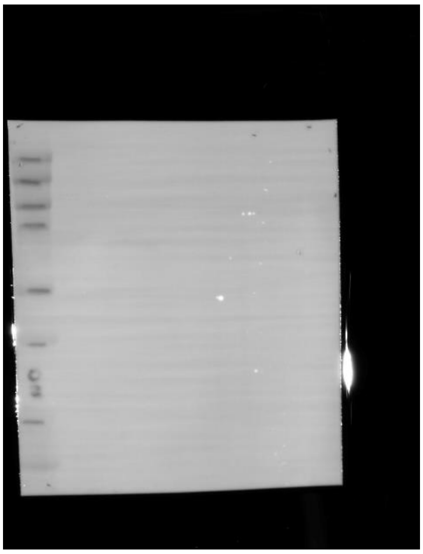

Supplementary Figure S12 continued.

A.

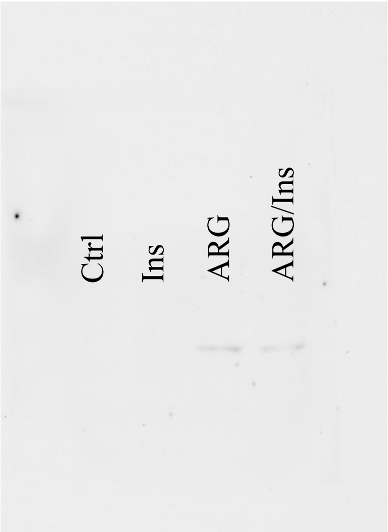

B.

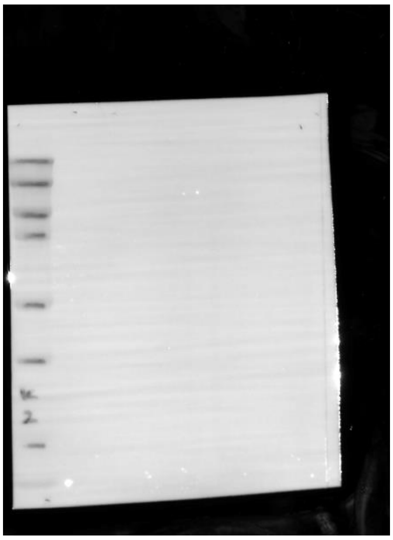

Supplementary Figure S13

C.

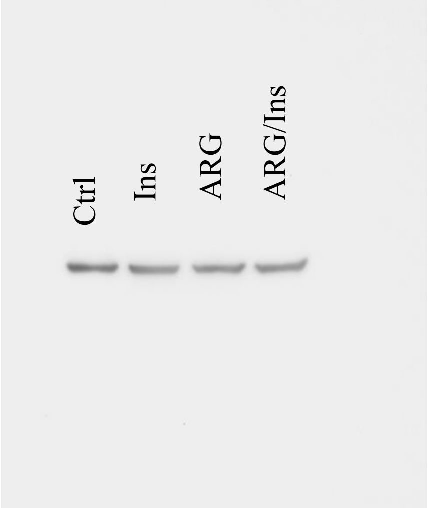

D.

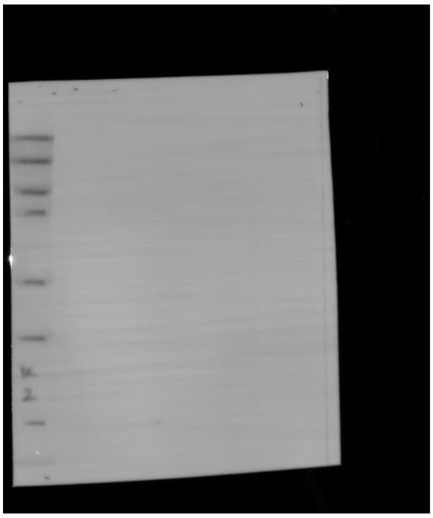

Supplementary Figure S13 continued.

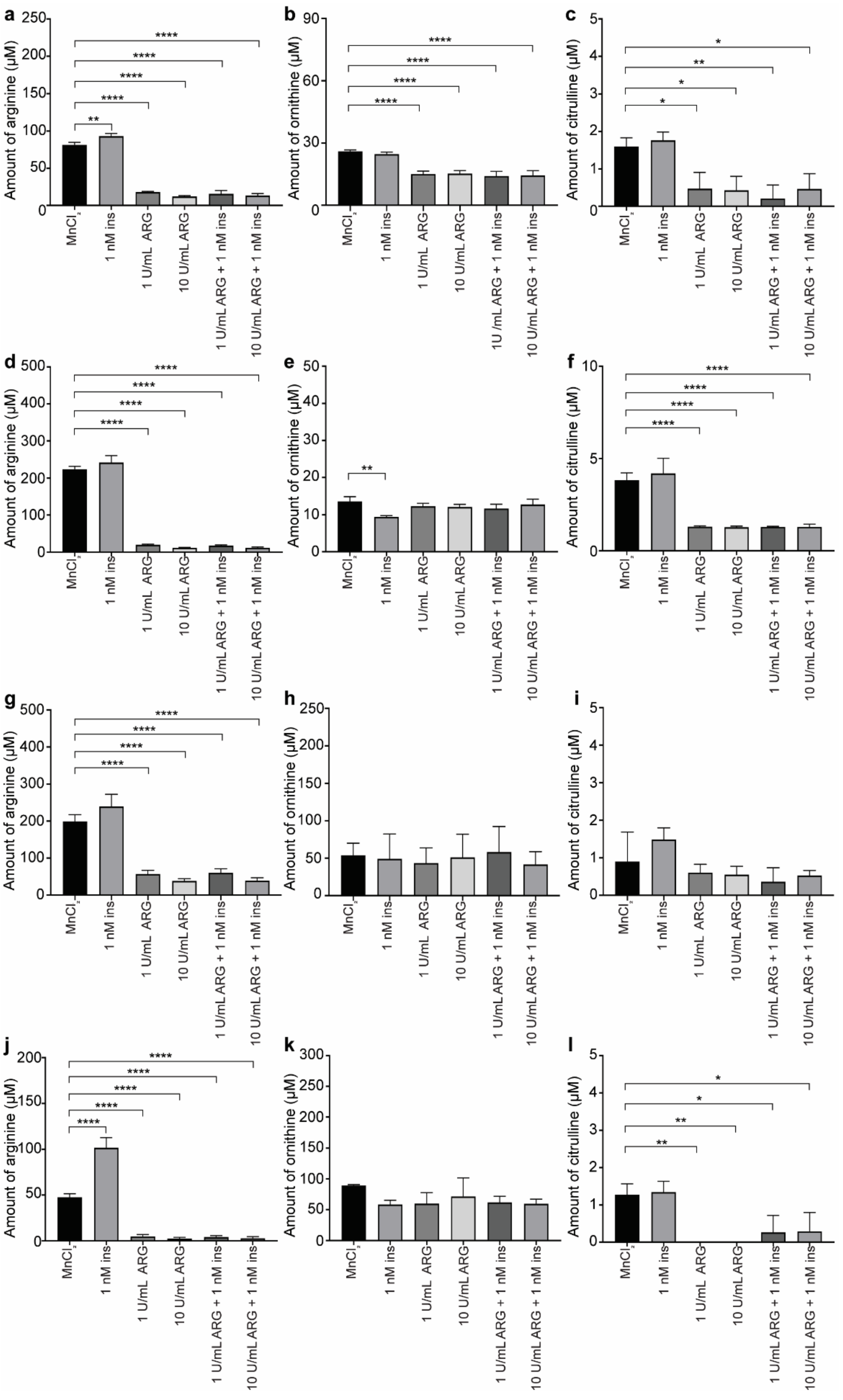

Supplementary Figure S14.

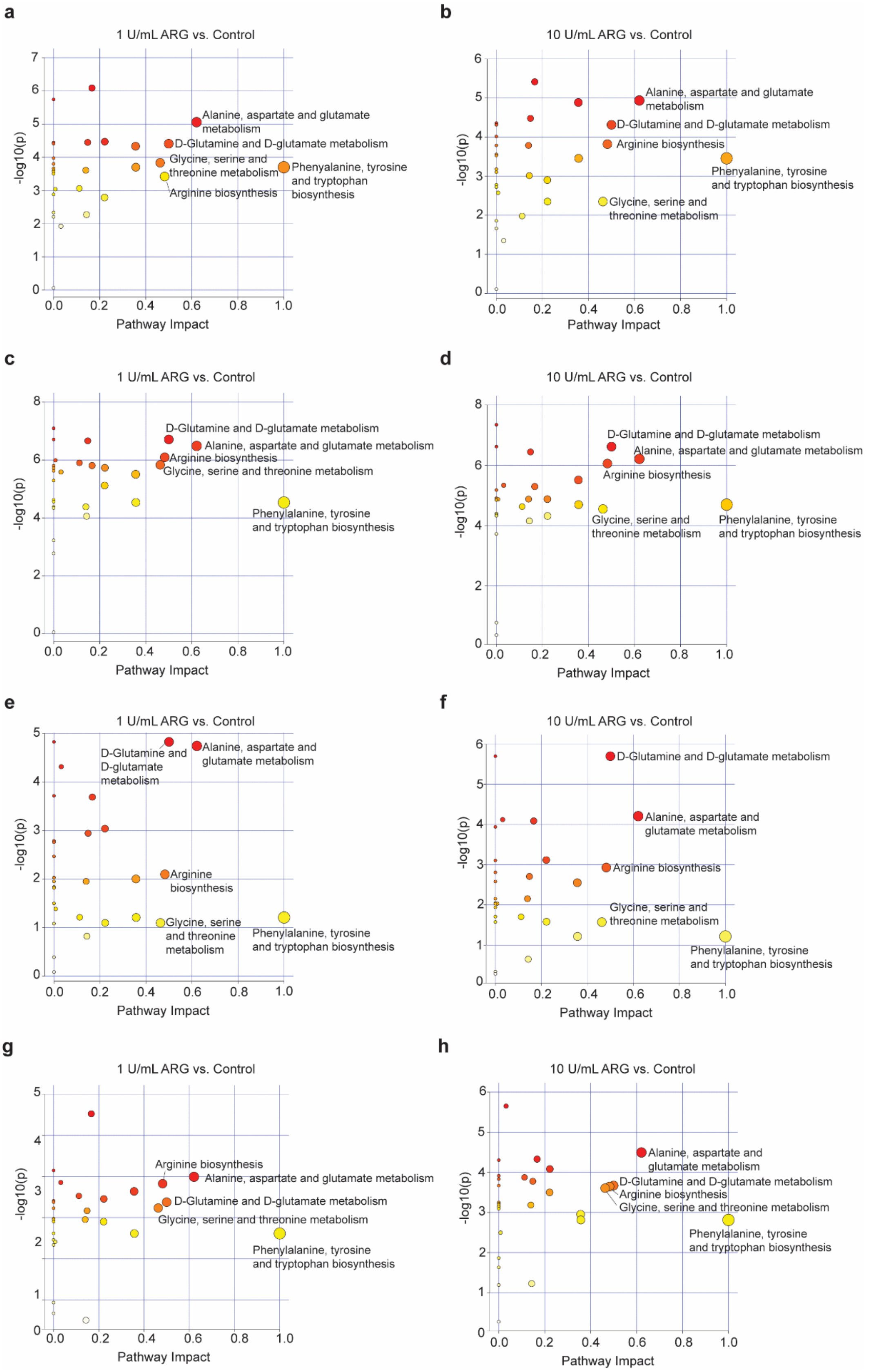

Supplementary Figure S15.

Supplementary Tables

Supplementary Table S1. A summary of volcano plot of differentially expressed metabolites in MDA-MB-231 cell lysates.

		MDA-MB-231		
**Amino Acid**	**KEGG ID**	**1 U/mL**	**1 U/mL + 1 nM**	**10 U/mL**
Aspartic acid	C00049	NS	NS	NS
Glutamic acid	C00025	NS	NS	NS
Cysteine	C00097	↓	↓	↓
Asparagine	C00152	↑	↑	↑
Serine	C00065	↑	↑	↑
Glutamine	C00064	↑	↑	↑
Histidine	C00135	NS	↓	↓
Glycine	C00037	NS	↓	↓
Threonine	C00188	NS	NS	NS
Arginine	C00062	↓	↓	↓
Alanine	C00041	↓	↓	↓
Gamma-Aminobutyric acid (GABA)	C00334	NS	NS	NS
Tyrosine	C00082	↓	↓	↓
Valine	C00183	↓	↓	↓
Methionine	C00073	NS	↓	↓
Tryptophan	C00078	NS	↓	↓
Phenylalanine	C00079	↓	↓	↓
Isoleucine	C00407	↓	↓	↓
Ornithine	C00077	↓	↓	↓
Leucine	C00123	↓	↓	↓
Lysine	C00047	↓	↓	↓
Proline	C00148	↓	↓	↓
Citrulline	C00327	↓	↓	↓

Arrow pointing up: increase in metabolite in treatment condition; arrow pointing down: decrease in metabolite in treatment condition; NS: not significant. Any differences observed between 1 U/mL ARG treatment and 1 U/mL ARG + 1 nM ins are highlighted in red.

Supplementary Table S2. A summary of volcano plot of differentially expressed metabolites in A549 cell lysates.

		**A549**		
**Amino Acid**	**KEGG ID**	**1 U/mL**	**1 U/mL + 1 nM**	**10 U/mL**
Aspartic acid	C00049	NS	NS	NS
Glutamic acid	C00025	↓	↓	↓
Cysteine	C00097	↓	↓	↓
Asparagine	C00152	↑	↑	↑
Serine	C00065	NS	NS	NS
Glutamine	C00064	↑	↑	↑
Histidine	C00135	↓	↓	↓
Glycine	C00037	↓	↓	↓
Threonine	C00188	↓	↓	↓
Arginine	C00062	↓	↓	↓
Alanine	C00041	↓	↓	↓
GABA	C00334	↓	↓	↓
Tyrosine	C00082	↓	↓	↓
Valine	C00183	↓	↓	↓
Methionine	C00073	NS	NS	↓
Tryptophan	C00078	↓	↓	↓
Phenylalanine	C00079	↓	↓	↓
Isoleucine	C00407	NS	↓	↓
Ornithine	C00077	NS	NS	NS
Leucine	C00123	NS	NS	↓
Lysine	C00047	↓	↓	↓
Proline	C00148	↓	↓	↓
Citrulline	C00327	↓	↓	↓

Arrow pointing up: increase in metabolite in treatment condition; arrow pointing down: decrease in metabolite in treatment condition; NS: not significant. Any differences observed between 1 U/mL ARG treatment and 1 U/mL ARG + 1 nM ins are highlighted in red.

Supplementary Table S3. A summary of volcano plot of differentially expressed metabolites in H1975 cell lysates.

		H1975		
**Amino Acid**	**KEGG ID**	**1 U/mL**	**1 U/mL + 1 nM**	**10 U/mL**
Aspartic acid	C00049	↓	↓	↓
Glutamic acid	C00025	↓	↓	↓
Cysteine	C00097	NS	NS	↓
Asparagine	C00152	↓	↓	↓
Serine	C00065	NS	NS	NS
Glutamine	C00064	↑	↑	↑
Histidine	C00135	↓	↓	↓
Glycine	C00037	↓	↓	↓
Threonine	C00188	NS	↑	NS
Arginine	C00062	↓	↓	↓
Alanine	C00041	↓	↓	↓
GABA	C00334	↓	NS	↓
Tyrosine	C00082	NS	NS	↓
Valine	C00183	↑	↑	NS
Methionine	C00073	↓	NS	↓
Tryptophan	C00078	NS	NS	NS
Phenylalanine	C00079	NS	↑	NS
Isoleucine	C00407	↓	↓	↓
Ornithine	C00077	NS	NS	NS
Leucine	C00123	NS	NS	↓
Lysine	C00047	NS	NS	NS
Proline	C00148	NS	NS	NS
Citrulline	C00327	NS	NS	NS

Arrow pointing up: increase in metabolite in treatment condition; arrow pointing down: decrease in metabolite in treatment condition; NS: not significant. Any differences observed between 1 U/mL ARG treatment and 1 U/mL ARG + 1 nM ins are highlighted in red.

Supplementary Table S4. A summary of volcano plot of differentially expressed metabolites in KURAMOCHI cell lysates.

		**KURAMOCHI**		
**Amino Acid**	**KEGG ID**	**1 U/mL**	**1 U/mL + 1 nM**	**10 U/mL**
Aspartic acid	C00049	↓	↓	↓
Glutamic acid	C00025	↓	↓	↓
Cysteine	C00097	↓	↓	↓
Asparagine	C00152	↓	↓	↓
Serine	C00065	NS	NS	NS
Glutamine	C00064	↓	↓	↓
Histidine	C00135	↓	↓	↓
Glycine	C00037	↓	↓	↓
Threonine	C00188	NS	NS	NS
Arginine	C00062	↓	↓	↓
Alanine	C00041	↓	↓	↓
GABA	C00334	↑	↑	↑
Tyrosine	C00082	↓	↓	↓
Valine	C00183	NS	NS	NS
Methionine	C00073	↓	↓	↓
Tryptophan	C00078	NS	NS	NS
Phenylalanine	C00079	NS	NS	NS
Isoleucine	C00407	NS	NS	NS
Ornithine	C00077	NS	NS	NS
Leucine	C00123	NS	NS	NS
Lysine	C00047	NS	NS	NS
Proline	C00148	↓	↓	↓
Citrulline	C00327	↓	↓	↓

Arrow pointing up: increase in metabolite in treatment condition; arrow pointing down: decrease in metabolite in treatment condition; NS: not significant. Any differences observed between 1 U/mL ARG treatment and 1 U/mL ARG + 1 nM ins are highlighted in red.

Supplementary Table S5. Metabolic pathway analysis of MDA-MB-231 when treated with 1 U/mL ARG.

**Pathway Name**	**Match Status**	**Raw *p***	**FDR**	**Impact**
Phenylalanine, tyrosine and tryptophan biosynthesis	2/4	1.99E-04	4.25E-04	1
Alanine, aspartate and glutamate metabolism	6/28	8.74E-06	9.33E-05	0.621
D-Glutamine and D-glutamate metabolism	2/6	3.90E-05	1.39E-04	0.5
Arginine biosynthesis	6/14	3.79E-04	5.52E-04	0.48223
Glycine, serine and threonine metabolism	4/33	1.46E-04	3.91E-04	0.46284
Phenylalanine metabolism	2/10	1.99E-04	4.25E-04	0.35714
Arginine and proline metabolism	5/38	4.64E-05	1.49E-04	0.35614
Cysteine and methionine metabolism	3/33	3.39E-05	1.39E-04	0.22222
Histidine metabolism	3/16	1.63E-03	2.00E-03	0.22131
Aminoacyl-tRNA biosynthesis	20/48	8.16E-07	2.61E-05	0.16667
Glyoxylate and dicarboxylate metabolism	4/32	3.56E-05	1.39E-04	0.14815
Tryptophan metabolism	1/41	5.34E-03	5.89E-03	0.14305
Tyrosine metabolism	1/42	2.46E-04	4.37E-04	0.13972
Glutathione metabolism	4/28	8.65E-04	1.20E-03	0.11182
Butanoate metabolism	2/15	1.19E-02	1.23E-02	0.03175
Primary bile acid biosynthesis	1/46	9.12E-04	1.22E-03	0.00758
Selenocompound metabolism	1/20	1.81E-06	2.89E-05	0
Purine metabolism	1/65	3.60E-05	1.39E-04	0
Pyrimidine metabolism	1/39	3.60E-05	1.39E-04	0
Nitrogen metabolism	2/6	3.90E-05	1.39E-04	0
Sphingolipid metabolism	1/21	1.05E-04	3.06E-04	0
Valine, leucine and isoleucine degradation	3/40	1.59E-04	3.91E-04	0
Valine, leucine and isoleucine biosynthesis	4/8	2.16E-04	4.31E-04	0
Ubiquinone and other terpenoid-quinone biosynthesis	1/9	2.46E-04	4.37E-04	0
Porphyrin and chlorophyll metabolism	2/30	2.73E-04	4.60E-04	0
Lysine degradation	1/25	3.16E-04	4.81E-04	0
Biotin metabolism	1/10	3.16E-04	4.81E-04	0
Pantothenate and CoA biosynthesis	3/19	1.31E-03	1.67E-03	0
Taurine and hypotaurine metabolism	1/8	4.60E-03	5.26E-03	0
Thiamine metabolism	1/7	4.60E-03	5.26E-03	0
beta-Alanine metabolism	2/21	6.21E-03	6.63E-03	0
Nicotinate and nicotinamide metabolism	1/15	8.62E-01	8.62E-01	0

The cut-off *p*-value is set at 0.1 FDR.

Supplementary Table S6. Metabolic pathway analysis of MDA-MB-231 when treated with 1 U/mL ARG and 1 nM insulin.

Pathway Name	Match Status	Raw *p*	FDR	Impact
Phenylalanine, tyrosine and tryptophan biosynthesis	2/4	3.68E-03	5.61E-03	1
Alanine, aspartate and glutamate metabolism	6/28	6.66E-06	2.13E-04	0.621
D-Glutamine and D-glutamate metabolism	2/6	9.26E-05	4.23E-04	0.5
Arginine biosynthesis	6/14	2.60E-04	9.26E-04	0.48223
Glycine, serine and threonine metabolism	4/33	1.36E-03	2.91E-03	0.46284
Phenylalanine metabolism	2/10	3.68E-03	5.61E-03	0.35714
Arginine and proline metabolism	5/38	9.22E-04	2.68E-03	0.35614
Cysteine and methionine metabolism	3/33	1.66E-04	6.63E-04	0.22222
Histidine metabolism	3/16	1.32E-02	1.59E-02	0.22131
Aminoacyl-tRNA biosynthesis	20/48	3.32E-05	4.23E-04	0.16667
Glyoxylate and dicarboxylate metabolism	4/32	6.59E-05	4.23E-04	0.14815
Tryptophan metabolism	1/41	1.73E-02	1.84E-02	0.14305
Tyrosine metabolism	1/42	3.18E-03	5.35E-03	0.13972
Glutathione metabolism	4/28	5.95E-03	8.65E-03	0.11182
Butanoate metabolism	2/15	1.47E-01	1.52E-01	0.03175
Primary bile acid biosynthesis	1/46	1.68E-02	1.84E-02	0.00758
Purine metabolism	1/65	8.46E-05	4.23E-04	0
Pyrimidine metabolism	1/39	8.46E-05	4.23E-04	0
Nitrogen metabolism	2/6	9.26E-05	4.23E-04	0
Selenocompound metabolism	1/20	5.40E-04	1.73E-03	0
Sphingolipid metabolism	1/21	1.04E-03	2.78E-03	0
Lysine degradation	1/25	1.26E-03	2.87E-03	0
Biotin metabolism	1/10	1.26E-03	2.87E-03	0
Valine, leucine and isoleucine degradation	3/40	1.76E-03	3.51E-03	0
Valine, leucine and isoleucine biosynthesis	4/8	2.83E-03	5.32E-03	0
Ubiquinone and other terpenoid-quinone biosynthesis	1/9	3.18E-03	5.35E-03	0
Pantothenate and CoA biosynthesis	3/19	7.85E-03	1.09E-02	0
Porphyrin and chlorophyll metabolism	2/30	1.28E-02	1.59E-02	0
Taurine and hypotaurine metabolism	1/8	1.34E-02	1.59E-02	0
Thiamine metabolism	1/7	1.34E-02	1.59E-02	0
beta-Alanine metabolism	2/21	1.63E-02	1.84E-02	0
Nicotinate and nicotinamide metabolism	1/15	9.00E-01	9.00E-01	0

The cut-off *p*-value is set at 0.1 FDR.

Supplementary Table S7. Metabolic pathway analysis of MDA-MB-231 when treated with 10 U/mL ARG.

**Pathway Name**	**Match Status**	**Raw *p***	**FDR**	**Impact**
Phenylalanine, tyrosine and tryptophan biosynthesis	2/4	3.50E-04	6.59E-04	1
Alanine, aspartate and glutamate metabolism	6/28	1.16E-05	1.39E-04	0.621
D-Glutamine and D-glutamate metabolism	2/6	4.85E-05	1.94E-04	0.5
Arginine biosynthesis	6/14	1.51E-04	4.36E-04	0.48223
Glycine, serine and threonine metabolism	4/33	4.48E-03	5.51E-03	0.46284
Phenylalanine metabolism	2/10	3.50E-04	6.59E-04	0.35714
Arginine and proline metabolism	5/38	1.30E-05	1.39E-04	0.35614
Cysteine and methionine metabolism	3/33	4.46E-03	5.51E-03	0.22222
Histidine metabolism	3/16	1.26E-03	1.92E-03	0.22131
Aminoacyl-tRNA biosynthesis	20/48	3.86E-06	1.23E-04	0.16667
Glyoxylate and dicarboxylate metabolism	4/32	3.32E-05	1.94E-04	0.14815
Tryptophan metabolism	1/41	9.73E-04	1.56E-03	0.14305
Tyrosine metabolism	1/42	1.63E-04	4.36E-04	0.13972
Glutathione metabolism	4/28	1.05E-02	1.24E-02	0.11182
Butanoate metabolism	2/15	4.47E-02	4.62E-02	0.03175
Primary bile acid biosynthesis	1/46	2.67E-03	3.56E-03	0.00758
Purine metabolism	1/65	4.40E-05	1.94E-04	0
Pyrimidine metabolism	1/39	4.40E-05	1.94E-04	0
Nitrogen metabolism	2/6	4.85E-05	1.94E-04	0
Selenocompound metabolism	1/20	9.68E-05	3.44E-04	0
Ubiquinone and other terpenoid-quinone biosynthesis	1/9	1.63E-04	4.36E-04	0
Lysine degradation	1/25	2.78E-04	6.32E-04	0
Biotin metabolism	1/10	2.78E-04	6.32E-04	0
Sphingolipid metabolism	1/21	2.96E-04	6.32E-04	0
Valine, leucine and isoleucine degradation	3/40	6.62E-04	1.18E-03	0
Valine, leucine and isoleucine biosynthesis	4/8	7.89E-04	1.33E-03	0
Porphyrin and chlorophyll metabolism	2/30	1.66E-03	2.42E-03	0
beta-Alanine metabolism	2/21	1.91E-03	2.66E-03	0
Pantothenate and CoA biosynthesis	3/19	1.39E-02	1.59E-02	0
Taurine and hypotaurine metabolism	1/8	2.19E-02	2.33E-02	0
Thiamine metabolism	1/7	2.19E-02	2.33E-02	0
Nicotinate and nicotinamide metabolism	1/15	7.80E-01	7.80E-01	0

The cut-off *p*-value is set at 0.1 FDR.

Supplementary Table S8. Metabolic pathway analysis of A549 when treated with 1 U/mL ARG.

**Pathway Name**	**Match Status**	**Raw *p***	**FDR**	**Impact**
Phenylalanine, tyrosine and tryptophan biosynthesis	2/4	2.93E-05	3.75E-05	1
Alanine, aspartate and glutamate metabolism	6/28	3.26E-07	1.74E-06	0.621
D-Glutamine and D-glutamate metabolism	2/6	1.96E-07	1.40E-06	0.5
Arginine biosynthesis	6/14	8.05E-07	3.68E-06	0.48223
Glycine, serine and threonine metabolism	4/33	1.48E-06	3.90E-06	0.46284
Phenylalanine metabolism	2/10	2.93E-05	3.75E-05	0.35714
Arginine and proline metabolism	5/38	3.13E-06	5.56E-06	0.35614
Cysteine and methionine metabolism	3/33	1.86E-06	4.08E-06	0.22222
Histidine metabolism	3/16	7.61E-06	1.22E-05	0.22131
Aminoacyl-tRNA biosynthesis	20/48	1.56E-06	3.90E-06	0.16667
Glyoxylate and dicarboxylate metabolism	4/32	2.18E-07	1.40E-06	0.14815
Tryptophan metabolism	1/41	8.76E-05	9.67E-05	0.14305
Tyrosine metabolism	1/42	4.13E-05	4.90E-05	0.13972
Glutathione metabolism	4/28	1.26E-06	3.90E-06	0.11182
Butanoate metabolism	2/15	2.60E-06	4.89E-06	0.03175
Primary bile acid biosynthesis	1/46	1.02E-06	3.90E-06	0.00758
Purine metabolism	1/65	8.06E-08	1.29E-06	0
Pyrimidine metabolism	1/39	8.06E-08	1.29E-06	0
Nitrogen metabolism	2/6	1.96E-07	1.40E-06	0
Taurine and hypotaurine metabolism	1/8	1.58E-06	3.90E-06	0
Thiamine metabolism	1/7	1.58E-06	3.90E-06	0
Porphyrin and chlorophyll metabolism	2/30	1.91E-06	4.08E-06	0
Pantothenate and CoA biosynthesis	3/19	2.36E-06	4.73E-06	0
Selenocompound metabolism	1/20	5.09E-06	8.58E-06	0
beta-Alanine metabolism	2/21	2.38E-05	3.63E-05	0
Lysine degradation	1/25	2.76E-05	3.75E-05	0
Biotin metabolism	1/10	2.76E-05	3.75E-05	0
Ubiquinone and other terpenoid-quinone biosynthesis	1/9	4.13E-05	4.90E-05	0
Valine, leucine and isoleucine biosynthesis	4/8	4.56E-05	5.22E-05	0
Valine, leucine and isoleucine degradation	3/40	5.95E-04	6.34E-04	0
Sphingolipid metabolism	1/21	1.67E-03	1.72E-03	0
Nicotinate and nicotinamide metabolism	1/15	8.90E-01	8.90E-01	0

The cut-off *p*-value is set at 0.1 FDR.

Supplementary Table S9. Metabolic pathway analysis of A549 when treated with 1 U/mL ARG and 1 nM insulin.

Pathway Name	Match Status	Raw *p*	FDR	Impact
Phenylalanine, tyrosine and tryptophan biosynthesis	2/4	5.50E-05	7.34E-05	1
Alanine, aspartate and glutamate metabolism	6/28	7.10E-07	3.29E-06	0.621
D-Glutamine and D-glutamate metabolism	2/6	6.71E-07	3.29E-06	0.5
Arginine biosynthesis	6/14	1.02E-06	3.64E-06	0.48223
Glycine, serine and threonine metabolism	4/33	2.69E-06	6.57E-06	0.46284
Phenylalanine metabolism	2/10	5.50E-05	7.34E-05	0.35714
Arginine and proline metabolism	5/38	4.39E-06	9.21E-06	0.35614
Cysteine and methionine metabolism	3/33	1.34E-06	4.29E-06	0.22222
Histidine metabolism	3/16	3.03E-05	4.85E-05	0.22131
Aminoacyl-tRNA biosynthesis	20/48	2.87E-06	6.57E-06	0.16667
Glyoxylate and dicarboxylate metabolism	4/32	6.48E-07	3.29E-06	0.14815
Tryptophan metabolism	1/41	3.07E-04	3.39E-04	0.14305
Tyrosine metabolism	1/42	3.62E-05	5.27E-05	0.13972
Glutathione metabolism	4/28	2.55E-06	6.57E-06	0.11182
Butanoate metabolism	2/15	4.60E-06	9.21E-06	0.03175
Primary bile acid biosynthesis	1/46	2.41E-05	4.06E-05	0.00758
Purine metabolism	1/65	4.93E-07	3.29E-06	0
Pyrimidine metabolism	1/39	4.93E-07	3.29E-06	0
Nitrogen metabolism	2/6	6.71E-07	3.29E-06	0
Taurine and hypotaurine metabolism	1/8	8.23E-07	3.29E-06	0
Thiamine metabolism	1/7	8.23E-07	3.29E-06	0
Pantothenate and CoA biosynthesis	3/19	1.66E-06	4.83E-06	0
Porphyrin and chlorophyll metabolism	2/30	1.81E-05	3.41E-05	0
Selenocompound metabolism	1/20	1.97E-05	3.51E-05	0
Ubiquinone and other terpenoid-quinone biosynthesis	1/9	3.62E-05	5.27E-05	0
beta-Alanine metabolism	2/21	8.30E-05	1.06E-04	0
Lysine degradation	1/25	9.94E-05	1.18E-04	0
Biotin metabolism	1/10	9.94E-05	1.18E-04	0
Valine, leucine and isoleucine biosynthesis	4/8	1.94E-04	2.22E-04	0
Valine, leucine and isoleucine degradation	3/40	1.23E-03	1.31E-03	0
Sphingolipid metabolism	1/21	2.98E-01	3.07E-01	0
Nicotinate and nicotinamide metabolism	1/15	8.61E-01	8.61E-01	0

The cut-off *p*-value is set at 0.1 FDR.

Supplementary Table S10. Metabolic pathway analysis of A549 when treated with 10 U/mL ARG.

**Pathway Name**	**Match Status**	**Raw *p***	**FDR**	**Impact**
Phenylalanine, tyrosine and tryptophan biosynthesis	2/4	2.03E-05	3.24E-05	1
Alanine, aspartate and glutamate metabolism	6/28	6.17E-07	3.29E-06	0.621
D-Glutamine and D-glutamate metabolism	2/6	2.42E-07	1.93E-06	0.5
Arginine biosynthesis	6/14	8.82E-07	4.03E-06	0.48223
Glycine, serine and threonine metabolism	4/33	2.82E-05	4.10E-05	0.46284
Phenylalanine metabolism	2/10	2.03E-05	3.24E-05	0.35714
Arginine and proline metabolism	5/38	3.09E-06	1.24E-05	0.35614
Cysteine and methionine metabolism	3/33	4.81E-05	5.50E-05	0.22222
Histidine metabolism	3/16	1.33E-05	2.53E-05	0.22131
Aminoacyl-tRNA biosynthesis	20/48	5.09E-06	1.63E-05	0.16667
Glyoxylate and dicarboxylate metabolism	4/32	3.63E-07	2.32E-06	0.14815
Tryptophan metabolism	1/41	7.03E-05	7.75E-05	0.14305
Tyrosine metabolism	1/42	1.33E-05	2.53E-05	0.13972
Glutathione metabolism	4/28	2.36E-05	3.60E-05	0.11182
Butanoate metabolism	2/15	4.62E-06	1.63E-05	0.03175
Primary bile acid biosynthesis	1/46	1.35E-05	2.53E-05	0.00758
Purine metabolism	1/65	4.54E-08	7.26E-07	0
Pyrimidine metabolism	1/39	4.54E-08	7.26E-07	0
Nitrogen metabolism	2/6	2.42E-07	1.93E-06	0
Porphyrin and chlorophyll metabolism	2/30	6.66E-06	1.94E-05	0
Selenocompound metabolism	1/20	1.24E-05	2.53E-05	0
Ubiquinone and other terpenoid-quinone biosynthesis	1/9	1.33E-05	2.53E-05	0
Lysine degradation	1/25	1.42E-05	2.53E-05	0
Biotin metabolism	1/10	1.42E-05	2.53E-05	0
beta-Alanine metabolism	2/21	4.03E-05	5.50E-05	0
Taurine and hypotaurine metabolism	1/8	4.35E-05	5.50E-05	0
Thiamine metabolism	1/7	4.35E-05	5.50E-05	0
Pantothenate and CoA biosynthesis	3/19	4.72E-05	5.50E-05	0
Valine, leucine and isoleucine biosynthesis	4/8	4.73E-05	5.50E-05	0
Valine, leucine and isoleucine degradation	3/40	1.90E-04	2.02E-04	0
Nicotinate and nicotinamide metabolism	1/15	1.66E-01	1.72E-01	0
Sphingolipid metabolism	1/21	4.30E-01	4.30E-01	0

The cut-off *p*-value is set at 0.1 FDR.

Supplementary Table S11. Metabolic pathway analysis of H1975 when treated with 1 U/mL ARG.

**Pathway Name**	**Match Status**	**Raw *p***	**FDR**	**Impact**
Phenylalanine, tyrosine and tryptophan biosynthesis	2/4	6.22E-02	8.29E-02	1
Alanine, aspartate and glutamate metabolism	6/28	1.81E-05	1.93E-04	0.621
D-Glutamine and D-glutamate metabolism	2/6	1.50E-05	1.93E-04	0.5
Arginine biosynthesis	6/14	8.03E-03	2.12E-02	0.48223
Glycine, serine and threonine metabolism	4/33	8.06E-02	9.52E-02	0.46284
Phenylalanine metabolism	2/10	6.22E-02	8.29E-02	0.35714
Arginine and proline metabolism	5/38	9.99E-03	2.12E-02	0.35614
Cysteine and methionine metabolism	3/33	8.05E-02	9.52E-02	0.22222
Histidine metabolism	3/16	9.17E-04	4.19E-03	0.22131
Aminoacyl-tRNA biosynthesis	20/48	2.06E-04	1.10E-03	0.16667
Glyoxylate and dicarboxylate metabolism	4/32	1.14E-03	4.58E-03	0.14815
Tryptophan metabolism	1/41	1.51E-01	1.67E-01	0.14305
Tyrosine metabolism	1/42	1.12E-02	2.12E-02	0.13972
Glutathione metabolism	4/28	6.17E-02	8.29E-02	0.11182
Butanoate metabolism	2/15	4.85E-05	3.88E-04	0.03175
Primary bile acid biosynthesis	1/46	4.15E-02	6.32E-02	0.00758
beta-Alanine metabolism	2/21	1.64E-03	5.60E-03	0
Biotin metabolism	1/10	4.10E-01	4.23E-01	0
Lysine degradation	1/25	4.10E-01	4.23E-01	0
Nicotinate and nicotinamide metabolism	1/15	3.42E-03	9.96E-03	0
Nitrogen metabolism	2/6	1.50E-05	1.93E-04	0
Pantothenate and CoA biosynthesis	3/19	3.19E-02	5.10E-02	0
Porphyrin and chlorophyll metabolism	2/30	1.75E-03	5.60E-03	0
Purine metabolism	1/65	9.37E-03	2.12E-02	0
Pyrimidine metabolism	1/39	9.37E-03	2.12E-02	0
Selenocompound metabolism	1/20	1.93E-04	1.10E-03	0
Sphingolipid metabolism	1/21	8.26E-01	8.26E-01	0
Taurine and hypotaurine metabolism	1/8	8.33E-02	9.52E-02	0
Thiamine metabolism	1/7	8.33E-02	9.52E-02	0
Ubiquinone and other terpenoid-quinone biosynthesis	1/9	1.12E-02	2.12E-02	0
Valine, leucine and isoleucine biosynthesis	4/8	1.46E-02	2.58E-02	0
Valine, leucine and isoleucine degradation	3/40	1.53E-02	2.58E-02	0

The cut-off *p*-value is set at 0.1 FDR.

Supplementary Table S12. Metabolic pathway analysis of H1975 when treated with 1 U/mL ARG and 1 nM insulin.

**Pathway Name**	**Match Status**	**Raw *p***	**FDR**	**Impact**
Phenylalanine, tyrosine and tryptophan biosynthesis	2/4	5.40E-02	8.65E-02	1
Alanine, aspartate and glutamate metabolism	6/28	4.47E-05	4.77E-04	0.621
D-Glutamine and D-glutamate metabolism	2/6	3.54E-06	5.67E-05	0.5
Arginine biosynthesis	6/14	7.94E-02	1.06E-01	0.48223
Glycine, serine and threonine metabolism	4/33	8.49E-02	1.07E-01	0.46284
Phenylalanine metabolism	2/10	5.40E-02	8.65E-02	0.35714
Arginine and proline metabolism	5/38	2.31E-02	4.63E-02	0.35614
Cysteine and methionine metabolism	3/33	9.37E-02	1.07E-01	0.22222
Histidine metabolism	3/16	1.54E-03	5.47E-03	0.22131
Aminoacyl-tRNA biosynthesis	20/48	6.06E-04	2.77E-03	0.16667
Glyoxylate and dicarboxylate metabolism	4/32	9.03E-05	5.78E-04	0.14815
Tryptophan metabolism	1/41	1.26E-01	1.40E-01	0.14305
Tyrosine metabolism	1/42	6.46E-02	9.39E-02	0.13972
Glutathione metabolism	4/28	7.41E-02	1.03E-01	0.11182
Butanoate metabolism	2/15	7.18E-05	5.74E-04	0.03175
Primary bile acid biosynthesis	1/46	4.81E-02	8.54E-02	0.00758
beta-Alanine metabolism	2/21	4.34E-03	1.16E-02	0
Biotin metabolism	1/10	6.92E-01	7.14E-01	0
Lysine degradation	1/25	6.92E-01	7.14E-01	0
Nicotinate and nicotinamide metabolism	1/15	7.02E-03	1.73E-02	0
Nitrogen metabolism	2/6	3.54E-06	5.67E-05	0
Pantothenate and CoA biosynthesis	3/19	4.33E-02	8.15E-02	0
Porphyrin and chlorophyll metabolism	2/30	1.27E-03	5.06E-03	0
Purine metabolism	1/65	3.85E-03	1.12E-02	0
Pyrimidine metabolism	1/39	3.85E-03	1.12E-02	0
Selenocompound metabolism	1/20	2.06E-04	1.10E-03	0
Sphingolipid metabolism	1/21	9.66E-01	9.66E-01	0
Taurine and hypotaurine metabolism	1/8	9.20E-02	1.07E-01	0
Thiamine metabolism	1/7	9.20E-02	1.07E-01	0
Ubiquinone and other terpenoid-quinone biosynthesis	1/9	6.46E-02	9.39E-02	0
Valine, leucine and isoleucine biosynthesis	4/8	1.59E-02	3.64E-02	0
Valine, leucine and isoleucine degradation	3/40	1.95E-02	4.16E-02	0

The cut-off *p*-value is set at 0.1 FDR.

Supplementary Table S13. Metabolic pathway analysis of H1975 when treated with 10 U/mL ARG.

**Pathway Name**	**Match Status**	**Raw *p***	**FDR**	**Impact**
Phenylalanine, tyrosine and tryptophan biosynthesis	2/4	6.05E-02	6.92E-02	1
Alanine, aspartate and glutamate metabolism	6/28	6.23E-05	5.25E-04	0.621
D-Glutamine and D-glutamate metabolism	2/6	2.02E-06	3.24E-05	0.5
Arginine biosynthesis	6/14	1.18E-03	4.18E-03	0.48223
Glycine, serine and threonine metabolism	4/33	2.67E-02	3.28E-02	0.46284
Phenylalanine metabolism	2/10	6.05E-02	6.92E-02	0.35714
Arginine and proline metabolism	5/38	2.80E-03	6.90E-03	0.35614
Cysteine and methionine metabolism	3/33	2.62E-02	3.28E-02	0.22222
Histidine metabolism	3/16	7.65E-04	3.14E-03	0.22131
Aminoacyl-tRNA biosynthesis	20/48	8.20E-05	5.25E-04	0.16667
Glyoxylate and dicarboxylate metabolism	4/32	1.96E-03	5.71E-03	0.14815
Tryptophan metabolism	1/41	2.22E-01	2.45E-01	0.14305
Tyrosine metabolism	1/42	7.00E-03	1.49E-02	0.13972
Glutathione metabolism	4/28	1.97E-02	2.87E-02	0.11182
Butanoate metabolism	2/15	7.60E-05	5.25E-04	0.03175
Primary bile acid biosynthesis	1/46	9.24E-03	1.66E-02	0.00758
beta-Alanine metabolism	2/21	1.55E-03	4.97E-03	0
Biotin metabolism	1/10	4.55E-01	4.70E-01	0
Lysine degradation	1/25	4.55E-01	4.70E-01	0
Nicotinate and nicotinamide metabolism	1/15	2.62E-03	6.90E-03	0
Nitrogen metabolism	2/6	2.02E-06	3.24E-05	0
Pantothenate and CoA biosynthesis	3/19	1.14E-02	1.93E-02	0
Porphyrin and chlorophyll metabolism	2/30	7.85E-04	3.14E-03	0
Purine metabolism	1/65	1.95E-02	2.87E-02	0
Pyrimidine metabolism	1/39	1.95E-02	2.87E-02	0
Selenocompound metabolism	1/20	1.15E-04	6.15E-04	0
Sphingolipid metabolism	1/21	5.14E-01	5.14E-01	0
Taurine and hypotaurine metabolism	1/8	2.64E-02	3.28E-02	0
Thiamine metabolism	1/7	2.64E-02	3.28E-02	0
Ubiquinone and other terpenoid-quinone biosynthesis	1/9	7.00E-03	1.49E-02	0
Valine, leucine and isoleucine biosynthesis	4/8	9.33E-03	1.66E-02	0
Valine, leucine and isoleucine degradation	3/40	8.93E-03	1.66E-02	0

The cut-off *p*-value is set at 0.1 FDR.

Supplementary Table S14. Metabolic pathway analysis of KURAMOCHI when treated with 1 U/mL ARG.

Pathway Name	Match Status	Raw *p*	FDR	Impact
Phenylalanine, tyrosine and tryptophan biosynthesis	2/4	2.43E-03	3.11E-03	1
Alanine, aspartate and glutamate metabolism	6/28	1.02E-04	9.56E-04	0.621
D-Glutamine and D-glutamate metabolism	2/6	4.21E-04	1.12E-03	0.5
Arginine biosynthesis	6/14	1.49E-04	9.56E-04	0.48223
Glycine, serine and threonine metabolism	4/33	5.82E-04	1.35E-03	0.46284
Phenylalanine metabolism	2/10	2.43E-03	3.11E-03	0.35714
Arginine and proline metabolism	5/38	2.30E-04	1.12E-03	0.35614
Cysteine and methionine metabolism	3/33	3.51E-04	1.12E-03	0.22222
Histidine metabolism	3/16	1.25E-03	1.82E-03	0.22131
Aminoacyl-tRNA biosynthesis	20/48	2.99E-06	9.57E-05	0.16667
Glyoxylate and dicarboxylate metabolism	4/32	6.79E-04	1.45E-03	0.14815
Tryptophan metabolism	1/41	3.13E-01	3.13E-01	0.14305
Tyrosine metabolism	1/42	1.11E-03	1.82E-03	0.13972
Glutathione metabolism	4/28	2.98E-04	1.12E-03	0.11182
Butanoate metabolism	2/15	1.40E-04	9.56E-04	0.03175
Primary bile acid biosynthesis	1/46	3.88E-03	4.60E-03	0.00758
beta-Alanine metabolism	2/21	2.41E-03	3.11E-03	0
Biotin metabolism	1/10	1.16E-01	1.23E-01	0
Lysine degradation	1/25	1.16E-01	1.23E-01	0
Nicotinate and nicotinamide metabolism	1/15	3.49E-03	4.29E-03	0
Nitrogen metabolism	2/6	4.21E-04	1.12E-03	0
Pantothenate and CoA biosynthesis	3/19	5.89E-04	1.35E-03	0
Porphyrin and chlorophyll metabolism	2/30	1.25E-03	1.82E-03	0
Purine metabolism	1/65	1.05E-03	1.82E-03	0
Pyrimidine metabolism	1/39	1.05E-03	1.82E-03	0
Selenocompound metabolism	1/20	7.14E-05	9.56E-04	0
Sphingolipid metabolism	1/21	2.12E-01	2.18E-01	0
Taurine and hypotaurine metabolism	1/8	3.91E-04	1.12E-03	0
Thiamine metabolism	1/7	3.91E-04	1.12E-03	0
Ubiquinone and other terpenoid-quinone biosynthesis	1/9	1.11E-03	1.82E-03	0
Valine, leucine and isoleucine biosynthesis	4/8	4.63E-03	5.29E-03	0
Valine, leucine and isoleucine degradation	3/40	1.24E-03	1.82E-03	0

The cut-off *p*-value is set at 0.1 FDR.

Supplementary Table S15. Metabolic pathway analysis of KURAMOCHI when treated with 1 U/mL ARG and 1 nM insulin.

**Pathway Name**	**Match Status**	**Raw *p***	**FDR**	**Impact**
Phenylalanine, tyrosine and tryptophan biosynthesis	2/4	6.93E-03	8.22E-03	1
Alanine, aspartate and glutamate metabolism	6/28	2.88E-05	3.20E-04	0.621
D-Glutamine and D-glutamate metabolism	2/6	1.28E-03	2.73E-03	0.5
Arginine biosynthesis	6/14	6.00E-05	3.20E-04	0.48223
Glycine, serine and threonine metabolism	4/33	1.42E-04	5.69E-04	0.46284
Phenylalanine metabolism	2/10	6.93E-03	8.22E-03	0.35714
Arginine and proline metabolism	5/38	3.61E-04	9.63E-04	0.35614
Cysteine and methionine metabolism	3/33	5.64E-05	3.20E-04	0.22222
Histidine metabolism	3/16	2.51E-03	4.46E-03	0.22131
Aminoacyl-tRNA biosynthesis	20/48	5.47E-05	3.20E-04	0.16667
Glyoxylate and dicarboxylate metabolism	4/32	1.25E-03	2.73E-03	0.14815
Tryptophan metabolism	1/41	5.31E-01	5.31E-01	0.14305
Tyrosine metabolism	1/42	3.32E-03	4.83E-03	0.13972
Glutathione metabolism	4/28	2.36E-04	7.57E-04	0.11182
Butanoate metabolism	2/15	2.85E-04	8.30E-04	0.03175
Primary bile acid biosynthesis	1/46	5.17E-03	6.62E-03	0.00758
beta-Alanine metabolism	2/21	4.09E-03	5.65E-03	0
Biotin metabolism	1/10	2.03E-01	2.10E-01	0
Lysine degradation	1/25	2.03E-01	2.10E-01	0
Nicotinate and nicotinamide metabolism	1/15	4.24E-03	5.65E-03	0
Nitrogen metabolism	2/6	1.28E-03	2.73E-03	0
Pantothenate and CoA biosynthesis	3/19	2.01E-04	7.14E-04	0
Porphyrin and chlorophyll metabolism	2/30	2.36E-03	4.45E-03	0
Purine metabolism	1/65	3.03E-03	4.83E-03	0
Pyrimidine metabolism	1/39	3.03E-03	4.83E-03	0
Selenocompound metabolism	1/20	1.22E-04	5.58E-04	0
Sphingolipid metabolism	1/21	1.42E-01	1.57E-01	0
Taurine and hypotaurine metabolism	1/8	4.26E-05	3.20E-04	0
Thiamine metabolism	1/7	4.26E-05	3.20E-04	0
Ubiquinone and other terpenoid-quinone biosynthesis	1/9	3.32E-03	4.83E-03	0
Valine, leucine and isoleucine biosynthesis	4/8	1.70E-02	1.94E-02	0
Valine, leucine and isoleucine degradation	3/40	1.62E-03	3.23E-03	0

The cut-off *p*-value is set at 0.1 FDR.

Supplementary Table S16. Metabolic pathway analysis of KURAMOCHI when treated with 10 U/mL ARG.

Pathway Name	Match Status	Raw *p*	FDR	Impact
Phenylalanine, tyrosine and tryptophan biosynthesis	2/4	1.54E-03	1.97E-03	1
Alanine, aspartate and glutamate metabolism	6/28	3.20E-05	3.97E-04	0.621
D-Glutamine and D-glutamate metabolism	2/6	2.13E-04	5.55E-04	0.5
Arginine biosynthesis	6/14	2.26E-04	5.55E-04	0.48223
Glycine, serine and threonine metabolism	4/33	2.48E-04	5.67E-04	0.46284
Phenylalanine metabolism	2/10	1.54E-03	1.97E-03	0.35714
Arginine and proline metabolism	5/38	1.11E-03	1.55E-03	0.35614
Cysteine and methionine metabolism	3/33	8.27E-05	5.23E-04	0.22222
Histidine metabolism	3/16	3.17E-04	6.76E-04	0.22131
Aminoacyl-tRNA biosynthesis	20/48	4.68E-05	3.97E-04	0.16667
Glyoxylate and dicarboxylate metabolism	4/32	1.67E-04	5.34E-04	0.14815
Tryptophan metabolism	1/41	5.91E-02	6.52E-02	0.14305
Tyrosine metabolism	1/42	6.53E-04	1.10E-03	0.13972
Glutathione metabolism	4/28	1.33E-04	5.23E-04	0.11182
Butanoate metabolism	2/15	2.24E-06	7.15E-05	0.03175
Primary bile acid biosynthesis	1/46	3.19E-03	3.92E-03	0.00758
beta-Alanine metabolism	2/21	6.00E-04	1.10E-03	0
Biotin metabolism	1/10	5.28E-01	5.28E-01	0
Lysine degradation	1/25	5.28E-01	5.28E-01	0
Nicotinate and nicotinamide metabolism	1/15	8.08E-04	1.18E-03	0
Nitrogen metabolism	2/6	2.13E-04	5.55E-04	0
Pantothenate and CoA biosynthesis	3/19	1.47E-04	5.23E-04	0
Porphyrin and chlorophyll metabolism	2/30	5.62E-04	1.10E-03	0
Purine metabolism	1/65	7.23E-04	1.10E-03	0
Pyrimidine metabolism	1/39	7.23E-04	1.10E-03	0
Selenocompound metabolism	1/20	4.97E-05	3.97E-04	0
Sphingolipid metabolism	1/21	6.42E-02	6.85E-02	0
Taurine and hypotaurine metabolism	1/8	1.22E-04	5.23E-04	0
Thiamine metabolism	1/7	1.22E-04	5.23E-04	0
Ubiquinone and other terpenoid-quinone biosynthesis	1/9	6.53E-04	1.10E-03	0
Valine, leucine and isoleucine biosynthesis	4/8	1.37E-02	1.62E-02	0
Valine, leucine and isoleucine degradation	3/40	2.33E-02	2.67E-02	0

The cut-off *p*-value is set at 0.1 FDR.
